# Distinct Stress‐Dependent Signatures of Cellular and Extracellular tRNA‐Derived Small RNAs

**DOI:** 10.1002/advs.202200829

**Published:** 2022-04-04

**Authors:** Guoping Li, Aidan C. Manning, Alex Bagi, Xinyu Yang, Priyanka Gokulnath, Michail Spanos, Jonathan Howard, Patricia P. Chan, Thadryan Sweeney, Robert Kitchen, Haobo Li, Brice D. Laurent, Sary F. Aranki, Maria I. Kontaridis, Louise C. Laurent, Kendall Van Keuren‐Jensen, Jochen Muehlschlegel, Todd M. Lowe, Saumya Das

**Affiliations:** ^1^ Cardiovascular Research Center Massachusetts General Hospital and Harvard Medical School Boston MA 02114 USA; ^2^ Department of Biomolecular Engineering Baskin School of Engineering University of California Santa Cruz Santa Cruz CA 95064 USA; ^3^ Fangshan Hospital of Beijing University of Traditional Chinese Medicine Beijing 102499 China; ^4^ Division of Cardiac Surgery Department of Surgery Brigham and Women's Hospital Harvard Medical School Boston MA 02115 USA; ^5^ Department of Biomedical Research and Translational Medicine Masonic Medical Research Institute Utica NY 13501 USA; ^6^ Department of Biological Chemistry and Molecular Pharmacology Harvard Medical School Boston MA 02115 USA; ^7^ Department of Medicine Division of Cardiology Beth Israel Deaconess Medical Center Harvard Medical School Boston MA 02215 USA; ^8^ Department of Obstetrics, Gynecology, and Reproductive Sciences University of California San Diego La Jolla CA 92093 USA; ^9^ Division of Neurogenomics The Translational Genomics Research Institute Phoenix AZ 85004 USA; ^10^ Department of Anesthesiology, Perioperative and Pain Medicine Brigham and Women's Hospital and Harvard Medical School Boston MA 02115 USA

**Keywords:** cellular stress, extracellular RNAs, noncoding small RNAs, RNA associated proteins, transfer RNA

## Abstract

The cellular response to stress is an important determinant of disease pathogenesis. Uncovering the molecular fingerprints of distinct stress responses may identify novel biomarkers and key signaling pathways for different diseases. Emerging evidence shows that transfer RNA‐derived small RNAs (tDRs) play pivotal roles in stress responses. However, RNA modifications present on tDRs are barriers to accurately quantifying tDRs using traditional small RNA sequencing. Here, AlkB‐facilitated methylation sequencing is used to generate a comprehensive landscape of cellular and extracellular tDR abundances in various cell types during different stress responses. Extracellular tDRs are found to have distinct fragmentation signatures from intracellular tDRs and these tDR signatures are better indicators of different stress responses than miRNAs. These distinct extracellular tDR fragmentation patterns and signatures are also observed in plasma from patients on cardiopulmonary bypass. It is additionally demonstrated that angiogenin and RNASE1 are themselves regulated by stressors and contribute to the stress‐modulated abundance of sub‐populations of cellular and extracellular tDRs. Finally, a sub‐population of extracellular tDRs is identified for which AGO2 appears to be required for their expression. Together, these findings provide a detailed profile of stress‐responsive tDRs and provide insight about tDR biogenesis and stability in response to cellular stressors.

## Graphical abstract

Using AlkB‐facilitated methylation sequencing, this work uncovers the distinct extracellular transfer RNA‐derived small RNA (tDR) fragmentation patterns from cellular tDRs and provides a detailed profile of stress‐responsive tDRs in a variety of cell culture systems and human patients. This work also provides insights about the biogenesis and stability of cellular and extracellular tDRs in response to cellular stressors.

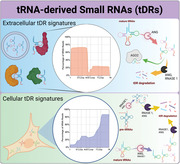

## 1 Introduction

Cells, including unicellular organisms, have evolved sophisticated sensing mechanisms and signal transduction systems for optimal response toward changes in environmental conditions (stress) to either ensure cell survival or alternatively elimination if the cell is unable to cope with the stress.^[^ ^1^, ^2^ ^]^ Examples of cellular stress responses include: DNA repair mechanisms triggered by DNA damage during ionizing radiation^[^ ^3^ ^]^; the unfolded protein response following heat shock or exposure to chemical toxins^[^ ^4^, ^5^ ^]^; activation of autophagy in response to nutritional deprivation^[^ ^6^ ^]^; induction of mitophagy to eliminate damaged mitochondria following hypoxic stress^[^ ^7^ ^]^; and adaptive responses to oxidative stress.^[^ ^8^, ^9^ ^]^ Increasing evidence has demonstrated that biological processes associated with stress responses play pivotal roles in normal development^[^ ^10^, ^11^ ^]^ and homeostasis,^[^ ^12^ ^]^ and failure of the adaptive stress response can lead to the onset or progression of various diseases.^[^ ^13^ ^]^ These cellular adaptations to stress involve a complex reorganization of the cellular gene expression program at the level of mRNA biogenesis,^[^ ^1^ ^]^ which is influenced by the dynamic regulation of the non‐coding RNA (ncRNA) transcriptome.

As one of the most abundant RNA species in cells, the canonical function of transfer RNAs (tRNAs) in decoding the genetic code during protein translation is well established.^[^ ^14^ ^]^ More recently, it has been shown that full‐length tRNA molecules are processed into smaller regulatory fragments, variously termed tRNA fragments and tRNA halves, or tRNA‐derived small RNAs (tDRs), by stress‐activated ribonucleases, including DICER, Angiogenin (ANG), ELAC2, and RNASE1, in a regimented manner.^[^ ^15^, ^16^ ^]^ tDRs can be grouped into numerous categories. Two of the most prominent types are 1) tRNA halves that are 30–50 nucleotides (nts) long generated by specific cleavage in or near the anticodon region, and 2) tRNA‐derived fragments that are usually 12–30 nts in length derived from cleavage of either mature or premature tRNAs at various positions.^[^ ^17^ ^]^ tDRs have been shown to play versatile roles in a variety of biological processes, including gene silencing, RNA stability, protein translation, RNA‐binding protein sequestration, epigenetic regulation, and ncRNA regulation.^[^ ^18^ ^]^ However, most existing studies exploring tDR biogenesis and regulation have used either hybridization‐based methods or conventional RNA sequencing techniques, which fail to capture a significant portion of the complex tDR pool. Specifically, the presence of tRNA base modifications can hinder the reverse transcription step during small RNA library generation, leading to inaccuracies in both the quantification of tDRs and base call accuracy, especially at the ends of sequences.^[^ ^19^ ^]^ Furthermore, the lack of a widely used, uniform nomenclature system, coupled with varied computational approaches to tDR read mapping, has led to difficulty in defining reproducible tDR signatures that can be easily compared between studies.^[^ ^20^, ^21^ ^]^ Advances in RNA sequencing methodologies, especially those customized for the RNA modifications commonly seen in tRNAs and tDRs, have helped to overcome the technical challenge of acquiring high‐quality data. These techniques, including AlkB‐facilitated methylation sequencing (ARM‐seq) and demethylase‐thermostable group II intron RT tRNA sequencing (DM‐tRNA‐seq), incorporate pretreatment of the input RNA samples with the AlkB enzyme to remove the modifications such as m^1^A, m^1^G, and m^3^C on tRNAs and tDRs to minimize stalling of reverse transcriptase at modified sites.^[^ ^19^, ^22^ ^]^

As mediators of intercellular communication, extracellular RNAs (exRNAs) have emerged as promising biomarkers for the diagnosis and prognosis of various diseases from minimally invasive liquid biopsies^[^ ^23^, ^24^ ^]^ In addition to high abundance in cells, tDRs also comprise a significant proportion of the extracellular RNAome.^[^ ^25^, ^26^ ^]^ This has been documented in multiple human biofluids, including urine, serum, plasma, saliva, and cerebrospinal fluid, and in cell‐conditioned medium^[^ ^24^, ^27^, ^28^ ^]^ Recent studies suggest a large proportion of extracellular tDRs in plasma or serum, notably the 5′‐tRNA halves of certain tRNA isodecoders are associated with ribonucleoproteins.^[^ ^24^ ^]^ There is also compelling evidence of tDRs associated with EVs in other biofluids and cell culture medium^[^ ^29^, ^30^ ^]^ While the presence of full‐length tRNAs within EVs and the site of tDR biogenesis remain topics currently under investigation^[^ ^31^, ^32^ ^]^ there does appear to be a correlation between the intracellular abundance of tDRs and their presence in EVs.^[^ ^33^ ^]^ Recently, several studies have indicated that circulating tDRs could serve as potential biomarkers for cancer diagnosis, prognosis after oncological therapies, monitoring cancer progression, liver fibrosis diagnosis, and distinguishing between subtypes of acute stroke.^[^ ^24^, ^34^ ^]^ Because conventional methods for detecting tDRs may underestimate the diversity and abundance of extracellular tDRs, building the stress‐specific extracellular tDR signatures using specialized tDR sequencing techniques promises to provide new markers of cellular processes associated with disease pathogenesis.

Here, we systematically profile matched cellular and extracellular tDRs expression using ARM‐seq in a variety of human and rat cells under three common stressors, including nutritional deprivation, hypoxia, and oxidative stress. We describe the unique fragmentation pattern of extracellular tDRs and the improved discrimination among different cellular stress responses using the extracellular tDR signatures. In preliminary studies, this distinct extracellular tDR fragmentation pattern and stress‐specific extracellular tDR signature was observed in plasma exRNAs from patients on cardiopulmonary bypass (CPB), which is a clinical condition involving acute metabolic and oxidative stress. We additionally demonstrate the critical roles of ANG and RNASE1 in the stress‐modulated cellular and extracellular tDR signatures and identify extracellular tDRs associated with AGO2 by generating cell lines with genetic ablation of ANG, RNASE1, and AGO2, respectively using CRISPR/Cas9 tools. These findings provide a comprehensive landscape of the dynamics, biogenesis, and stability of cellular and extracellular tDR expression induced by common stressors and demonstrate the potential of extracellular tDR signatures as possible biomarkers for a variety of human diseases.

## 2 Results

### 2.1 The Establishment of In Vitro Stress Response Models for tDR Expression Profiling

**Figure 1:**
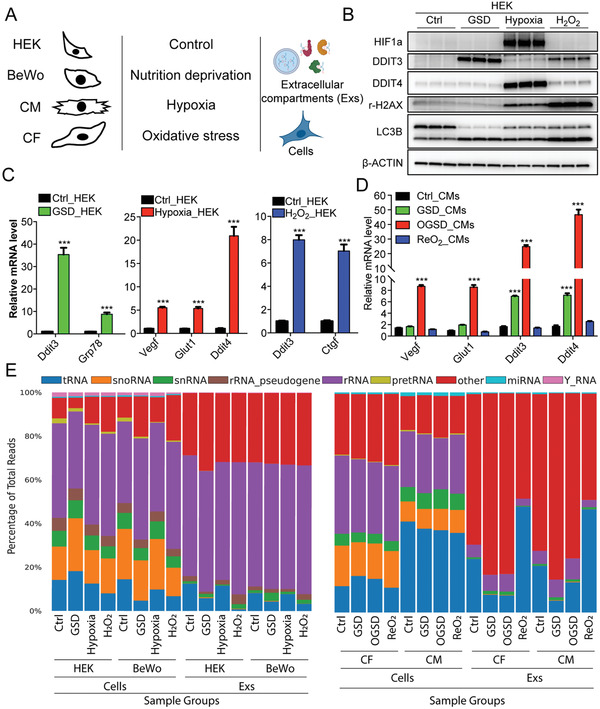
ARM‐seq reveals robust information about the cellular and extracellular tDR expression profiles during stress response. A) Schematic representation of the samples collected for ARM‐seq. B) Western blot validation of different stress responses in HEK cells: GSD induced the expression of DDIT3 and the cleavage of LC3B; hypoxia stabilized HIF1a protein and induced DDIT4 expression; H_2_O_2_ induced DDIT3 expression and increased the levels of r‐H2AX. C) qPCR validation of different stress responses in HEK cells: GSD increased the mRNA levels of Ddit3 and Grp78; hypoxia activated the transcription of Vegf, Glut1, and Ddit4; H_2_O_2_ elevated the transcript levels of Ddit4 and Ctgf. D) qPCR validation of different stress responses in CM cells: GSD activated the expression level of Ddit3 and Ddit4; OGSD induced the angiogenesis‐related genes, including Vegf and Glut1; ReO_2_ restored the levels of these genes induced by OGSD. E) ARM‐seq detects decent amount of tDRs reads from both cells and Exs in human and rat samples; “others” includes mRNA, lincRNAs, and all of the other RNA species. Data are shown as means ± SEM of at least three independent experiments. The unpaired two‐tailed Student's *t*‐test was used in (C,D). ***p < 0.001 versus the control group.

To systematically profile the molecular signatures of tDRs under different stress conditions, we focused on three common perturbations: nutritional deprivation (glucose and serum deprivation, GSD), hypoxia, and oxidative stress. To enhance the rigor of the study and determine consistent signatures across different cell types, these three perturbations were induced in four different in vitro cell culture systems individually, including human embryonic kidney cells—HEK293 (HEK), human choriocarcinoma cells—BeWo, primary neonatal rat ventricular cardiomyocytes (CM), and primary neonatal rat ventricular cardiac fibroblasts (CF) (**Figure** **1**A).

HEK and BeWo cells were exposed to each stressor for 24 h. Along with cellular RNAs and proteins, exRNAs associated with extracellular compartments (Exs) were also isolated from the conditional cell culture medium using an exoRNeasy kit. Cellular stress responses were confirmed by western blot and qPCR. As expected, GSD significantly activated autophagy and the expression of Ddit3 and Grp78^[^ ^35^ ^]^ (Figure 1B,C; Figure S1A,B, Supporting Information). Hypoxia stabilized the HIF1a protein and transactivated the expression of Vegf, Glut1, and Ddit4^[^ ^36^ ^]^ (Figure 1B,D; Figure S1A,C, Supporting Information). Oxidative stress, induced by exposure to hydrogen peroxide (H_2_O_2_), significantly induced DNA damage and activated the expression of Ddit3 and Ctgf^[^ ^37^ ^]^ (Figure 1B,E; Figure S1A,D, Supporting Information). To mimic the cardiac ischemia/reperfusion injury, CM and CF were exposed to a condition with oxygen, glucose, and serum deprivation (OGSD), for 5 h and reoxygenated for an additional 24 h.^[^ ^38^ ^]^ Strikingly, there was a rapid response upon GSD or OGSD treatment, and reoxygenation (ReO_2_) eliminated most of the OGSD‐responsive genes in both CMs and CFs (Figure 1D; Figure S1E, Supporting Information). These data provide strong support for our established in vitro stress response models to be used to profile stress‐dependent cellular and extracellular tDR signatures.

### 2.2 ARM‐seq Greatly Increases the Abundance and Diversity of tDRs Detected

Previously, we developed the ARM‐seq platform, which improves the quantification of methylated tDR abundance.^[^ ^19^ ^]^ To verify ARM‐seq improves the quantification of tDRs, small RNAs isolated from HEK cells under different stressors were treated with or without purified recombinant His‐AlkB and then subjected to deep sequencing after small RNA library preparation. Mapping, annotation, and analysis confirmed that AlkB treatment dramatically increases the proportion of small RNA sequencing reads from tRNA genes (Figure S1F, Supporting Information). Notably, reads after AlkB treatment extend through the ubiquitously modified m^1^A at position 58 of most tRNAs (Figure S1G, Supporting Information). Most importantly, ARM‐seq provides an abundance of high‐resolution information about the dynamic regulation of tDRs during the stress response, including nutrient deprivation‐elevated internal fragments of tRNA‐Phe‐GAA‐2/3 and hypoxia‐induced 5′tDRs of tRNA‐Asp‐GTC‐2 (Figure S1H, Supporting Information), which were nearly undetectable by standard small RNA‐seq. As a result, we conclude that ARM‐seq performed using our purified recombinant His‐AlkB works efficiently and facilitates the accurate and robust quantification of tDRs.

### 2.3 Overview of Intracellular and Extracellular tDR Expression During the Stress Response

A total of 96 bar‐coded small RNA libraries, including three independent replicates for each of the 32 conditions (4 cell types × 4 stressors × 2 sample types) (Figure 1A), were prepared and sequenced from our in vitro stress response models using ARM‐seq. Approximately 95.65% of the reads from human samples mapped to the human genome, and 95.57% of the reads from rat samples were mapped to the rat genome. Within these, a significant proportion of the reads from both cells and Exs correspond to tRNAs (Figure 1E). Interestingly, almost 35% of sequencing reads were aligned to tRNA genes in CMs. In comparison, this proportion was about 15% in other cell types, which could indicate CMs as having a higher relative tDR expression (Figure 1E). In addition, ReO_2_ dramatically increased the proportion of tRNA reads in both CF and CM‐derived Exs from ≈25% to ≈50% (Figure 1E), which suggests an important regulation of extracellular tDRs expression in the heart cells after ischemia/reperfusion. tRNA isotypes exhibited similar distributions amongst the different cell types, except for the CMs, which had a higher proportion of tRNA‐Phe (Figure S2A,B, Supporting Information). The extracellular tRNA isotype distributions were also similar among the Exs derived from different cell types but had higher proportions of tRNA‐Glu, tRNA‐Gly, and tRNA‐Pro, and lower proportions of tRNA‐Arg, tRNA‐Gln, and tRNA‐His, when compared with cellular tRNA isotypes (Figure S2A,B, Supporting Information).

### 2.4 Intracellular and Extracellular tDRs have Distinct Fragmentation Signatures

**Figure 2:**
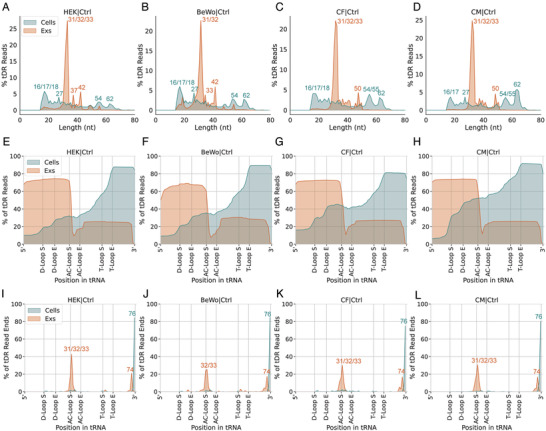
Extracellular tDRs show distinct fragmentation signatures from cellular tDRs. Extracellular tDRs are predominantly 31–33 nts in length in all profiled cell types, including A) HEK, B) BeWo, C) CF, and D) CM. Extracellular tDRs are predominantly tRNA halves and derived from both ends of tRNA genes while intracellular tDRs are mainly generated from 3′ end of tRNAs with various lengths in all cell types tested, including E) HEK, F) BeWo, G) CF, and H) CM. Extracellular tDRs end at either position 33 or 74 while most of the intracellular tDRs end at position 76 in all examined cell types, including I) HEK, J) BeWo, K) CF, and L) CM cells.

Increasing evidence has shown that tDRs are a major component of exRNAs and account for the majority of mapped reads in many biofluids tested^[^ ^27^, ^39^ ^]^ However, the fragmentation profiles of intracellular and extracellular tDRs have not been systematically studied. Here, we first analyzed the length distribution of tDRs amongst the 96 samples. Strikingly, the extracellular tDRs were predominantly 31–33 nts in length across the species, cell types, and stressors. Traditionally, tDRs of this length correspond to tRNA‐derived halves.^[^ ^40^ ^]^ In contrast, the intracellular tDRs had a wide range of length distribution (**Figure** **2**A–D; Figure S3A–L, Supporting Information), consistent with prior work.^[^ ^41^ ^]^ This predominance for a specific length appeared to be specific to tDRs and was not observed across other small exRNA types (Figure S4A–P, Supporting Information). To further confirm the subtypes of these specific extracellular tDRs, the per‐base read coverage across each position of the mature tRNA isodecoder was analyzed. We observe that around 70% of the extracellular tDRs correspond to 5′ halves of tRNAs, and about 20% derive from the 3′ end of the transcript, whereas the intracellular tDRs are derived explicitly from the 3′ end of tRNAs (Figure 2E–H; Figure S5A–L, Supporting Information). Looking at the termination position of read ends, we further confirmed that the extracellular 5′tDRs end at the anticodon loop, which usually generates 5′ tRNA halves (Figure 2I–L; Figure S6A–I, Supporting Information). Notably, almost all the extracellular tDRs derived from the 3′ end of tRNAs had a trimmed CCA tail ending with a single cytosine, while most of the intracellular 3′tDRs had an intact CCA tail (Figure 2I–L; Figure S6A–L, Supporting Information), suggesting that extracellular 3′tDRs may have a specific biogenesis mechanism that is distinct from their intracellular counterparts. Alternatively, the trimmed CCA tail of the 3′tDRs may confer them increased stability in the Exs, leading to increased relative expression. In summary, these data strongly demonstrate that tDRs have distinct fragmentation signatures between cells and Exs.

### 2.5 Extracellular tDR Expression Profiles Provide Better Discrimination between Different Stress Responses Compared to miRNAs

**Figure 3:**
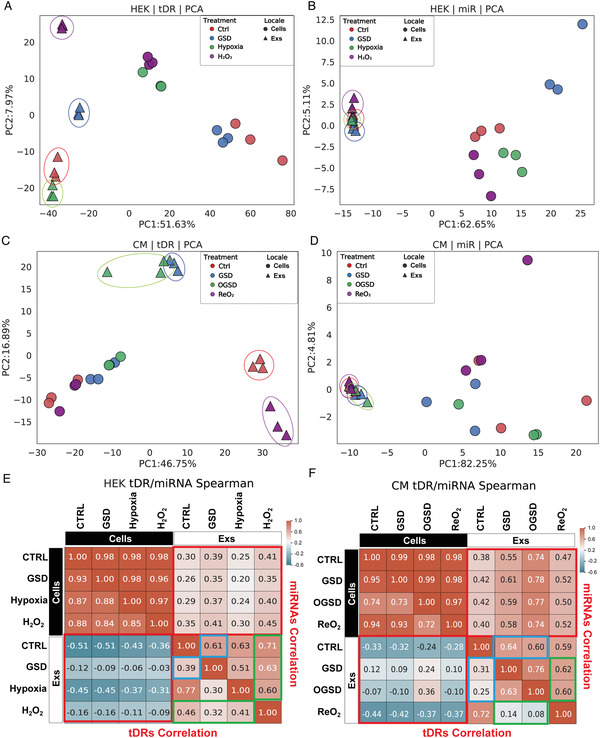
Extracellular tDR expression landscapes provide better discrimination between different stress responses compared to miRNAs. PCA analysis of A) tDR profiles provides better resolution to distinguish EV samples (circled) derived from HEK cells after different stress treatments than B) miRNA profiles. PCA analysis of C) tDR profiles provides better resolution to distinguish EV samples (circled) derived from CM cells after different stress treatments than D) miRNA profiles. E) Heatmaps of correlation coefficients (Spearman) for tDR class (left bottom) shows larger variance among different samples than miRNA class (right top) in HEK cells. F) Heatmaps of correlation coefficients (Spearman) for tDR class (left bottom) shows larger variance among different samples than miRNA class (right top) in CM cells. In (E,F), red boxes show the difference between intracellular samples and extracellular samples, blue boxes indicate the difference between each stressor and control group in Exs samples, and green boxes present the difference between different stressors in Exs samples.

Extracellular miRNA expression has been extensively studied to identify biomarkers for the diagnosis and prognosis of diseases,^[^ ^42^ ^]^ whereas our knowledge of extracellular tDRs is still emerging. To evaluate the capability of tDRs to distinguish between different stress responses, we assessed the differences in the abundance levels of different small RNA species, including tDRs and miRNAs, across all profiled samples from each cell type by performing principal component analysis (PCA). Interestingly, upon visual interrogation of the 24 samples from HEK cells based on tDR signatures, cellular samples subjected to different treatments were largely overlapping, whereas different stressor‐induced EV tDR profiles were clearly distinguished from each other (**Figure** **3**A). In contrast, clustering based on extracellular miRNA expression profiles did not separate samples according to stressor exposure, while the cellular miRNA expression signatures were able to distinguish between them (Figure 3B). The unique property of extracellular tDR expression profiles to discriminate different stress responses was also demonstrated in BeWo (Figure S7A,B, Supporting Information), CM (Figure 3C,D), and CF (Figure S7C,D, Supporting Information) cells.

We performed dimensionality reduction via Uniform Manifold Approximation and Projection (UMAP) to further support these findings. As expected, the UMAP for tDR signatures clearly delineated each stressor‐exposed exRNA sample from exRNA samples exposed to other stressors in each profiled cell type. In contrast, the extracellular miRNA expression profiles had a limited capacity to discriminate among different stress treatments (Figure S8A–H, Supporting Information). Finally, similar to the pattern observed for miRNA expression, tDR expression was highly correlated across the four different cellular samples, but showed poor correlation between cellular and extracellular samples (Figure 3E,F; Figure S9A,B, Supporting Information), indicating significant differences between cellular and extracellular small RNA expression profiles. The tDR expression profiles appeared to be more divergent between the extracellular and cellular compartments and between the different stress responses than the corresponding miRNA expression profiles (Figure 3E,F; Figure S9A,B, Supporting Information). Taken together, these data strongly suggest that the extracellular tDRs expression signatures are not simply a reflection of the stoichiometry of cellular tDR expression and provide improved discrimination between different stress responses than miRNA signatures.

### 2.6 Nutritional Deprivation‐Shaped Cellular and Extracellular tDR Signatures

Stress, especially amino acid starvation, has been shown to induce dynamic expression of tDRs.^[^ ^20^ ^]^ However, the abundance and diversity of tDRs were previously underestimated due to the technical shortcomings of conventional small RNA sequencing. To systematically study the regulation of tDRs after nutrition deprivation, we assessed differentially expressed (DE) cellular and extracellular tDRs in response to nutritional deprivation in all four cell types. By tracking the expression of each tDR in HEK cells or in Exs with or without GSD treatment, we again noted the distinct expression profiles of cellular and extracellular tDRs (Figure S10A, Supporting Information), confirming our previous conclusion that extracellular tDRs do not simply reflect the stoichiometry of cellular tDRs. Strikingly, extracellular tDRs were far more dynamically regulated after GSD treatment when compared with cellular tDRs (Figure S10A, Supporting Information). In contrast, there were fewer extracellular miRNAs expressed with or without GSD treatment, although the cellular miRNA expression levels were comparable to the level of tDRs (Figure S10B, Supporting Information). These findings were also noted in BeWo (Figure S10C,D, Supporting Information), CM (Figure S10E,F, Supporting Information), and CF cells (Figure S10G,H, Supporting Information). Together, our results demonstrate that the biogenesis or stability of extracellular tDRs (and to a lesser extent, cellular tDRs) is dynamically regulated by the cellular response to nutrient deprivation and may provide a more sensitive marker of metabolic stress than miRNAs.

In HEK cells, differential expression analysis revealed 312 upregulated and 254 downregulated cellular tDRs and 1318 upregulated and 1949 downregulated extracellular tDRs after GSD treatment (Table S1, Supporting Information). To complement our sequencing data, we validated the most abundant DE tDRs, including downregulated cellular tDR‐T1‐T31‐Arg‐CCT‐2‐1, downregulated extracellular tDR‐37:74‐Asp‐GTC‐2‐M2, and upregulated extracellular tDR‐1:36‐Asp‐GTC‐2, using northern blots (Figure S11A–E, Supporting Information). In BeWo cells, there were 121 upregulated and 1232 downregulated cellular tDRs and 624 upregulated and 1541 downregulated extracellular tDRs after GSD treatment (Table S1, Supporting Information). Notably, 40 DE cellular tDRs were common to both HEK and BeWo cells with nutritional deprivation, which are mainly derived from tRNA‐Lys and tRNA‐Glu (Figure S11F, Supporting Information). As opposed to the cellular tDRs, there were 1058 common DE extracellular tDRs upon GSD treatment, of which more than 60% were derived from tRNA‐Glu, tRNA‐Pro, tRNA‐Ser, and tRNA‐Gly (Figure S11F, Supporting Information). The top five significantly differentially abundant cellular tDRs and top nine extracellular tDRs are listed in Figure S11G, Supporting Information. Interestingly, six of these tDRs were differentially abundant in both cells and Exs in response to GSD for both cell types, including upregulated tDR‐37:75‐Glu‐TTC‐1‐M2 and downregulated tDR‐1:32‐Glu‐TTC‐2 (Figure S11F,G, Supporting Information).

In the rodent primary cells cultured in GSD, differential expression analysis identified 182 DE cellular tDRs in neonatal rat CFs, and only 36 changed cellular tDRs in CMs, of which nine were shared between the two cell types (Figure S11H, Table S2, Supporting Information). In contrast, there were 4025 and 3500 DE extracellular tDRs for CFs and CMs after GSD, respectively, of which more than 30% of these were upregulated (Figure S11H, Table S2, Supporting Information). Of the 2631 extracellular tDRs that were significantly different in both cell types, more than 50% were derived from tRNA‐Glu, tRNA‐Ser, tRNA‐Pro, and tRNA‐Gly, which is similar to the GSD‐modulated human extracellular tDRs (Figure S11H, Supporting Information). The top four significantly differentially abundant tDRs and top ten significant extracellular tDRs associated with both cell types are enumerated in Figure S11I, Supporting Information. Interestingly, we found several conserved extracellular tDRs among rat and human species that were commonly regulated by GSD across all four cell types, including the upregulated tDR‐1:36‐Gly‐CCC‐1 and tDR‐1:36‐Asp‐GTC‐2‐M2, and the downregulated tDR‐1:33‐Pro‐AGG‐1‐M5 and tDR‐42:74‐Ser‐GCT‐1 (Tables S1,S2, Supporting Information). These data suggest that there may be a “universal” extracellular tDR signature of nutritional deprivation.

### 2.7 Hypoxia‐Shaped Cellular and Extracellular tDR Signatures

The cellular response to hypoxia plays a key role in the pathogenesis of many diseases, including myocardial ischemia, metabolic disorders, chronic heart and kidney diseases, and reproductive diseases.^[^ ^43^ ^]^ To comprehensively profile the tDR signature corresponding to the cellular response to hypoxia, we compared the abundance levels of cellular and extracellular tDRs between normoxic and hypoxic conditions across the four cell types. Consistent with our findings for nutritional deprivation, RNA expression tracking plots showed more dramatic changes in the expression of extracellular tDR expression than intracellular tDR expression and more pronounced changes in tDRs than miRNAs, in all profiled cell types upon hypoxia treatment (Figure S12A–H, Supporting Information).

We observed 743 DE cellular tDRs and 1463 DE extracellular tDRs in HEK cells (Table S3, Supporting Information). We also verified the most significant DE tDRs, including decreased cellular tDR‐T1:T20‐Ser‐TGA‐1‐1, increased cellular tDR‐1:32‐Asp‐GTC‐2, and upregulated extracellular tDR‐39:74‐Glu‐TTC‐2, using northern blots (Figure S13A–H, Supporting Information). Unlike the nutritional deprivation response, alternations in tDRs abundance in response to hypoxia were less pronounced in BeWo cells with 271 DE cellular tDRs and 173 DE extracellular tDRs (Table S3, Figure S13I, Supporting Information). We noted an increase of tDR‐1:34‐Gly‐GCC‐1, which was previously reported to be induced by hypoxia in triple‐negative breast cancer cells,^[^ ^44^ ^]^ in hypoxia‐treated BeWo and HEK cells (Table S3, Supporting Information). Strikingly, 117 out of 121 cellular tDRs that were altered by hypoxia in both HEK and BeWo cells were downregulated and more than 50% of them are derived from tRNA‐Arg, tRNA‐Leu, and tRNA‐Lys (Figure S13I, Table S3, Supporting Information). There were 71 hypoxia‐regulated extracellular tDRs common to HEK and BeWo cells, among which 65% of them are derived from tRNA‐Glu (Figure S13I, Table S3, Supporting Information). The top four significantly differentially abundant cellular tDRs and top eight significant extracellular tDRs associated with both cell types are listed in Figure S13J, Supporting Information.

The differential expression analysis revealed more pronounced changes in extracellular tDRs in OGSD‐treated rat CF and CM samples, with 3931 DE extracellular tDRs associated with CF and 5259 DE tDRs associated with CMs (Figure S13K, Table S4, Supporting Information). Half of the 2456 extracellular tDRs that were changed by OGSD in both cell types were derived from tRNA‐Glu, tRNA‐Ser, tRNA‐Pro, and tRNA‐Gly (Figure S13K, Supporting Information), which is similar to the GSD treatments in both human and rat cells. However, we still identified 431 extracellular tDRs are altered explicitly by OGSD treatment but not GSD treatment (Tables S2,S4, Supporting Information). In the cells, there were 2502 DE tDRs in CM cells but only 228 tDRs were changed in CF cells after OGSD treatment (Figure S13K, Supporting Information). Of them, 51 are mainly derived from tRNA‐Glu, tRNA‐Asp, and tRNA‐Ser, which were significantly regulated by OGSD treatment in both CF and CM cells (Figure S13K, Supporting Information). The top five significantly altered cellular tDRs and top ten DE extracellular tDRs are enumerated in Figure S13L, Supporting Information. Notably, we also found some conserved extracellular tDRs among human and rat species that were downregulated by hypoxia in the Exs derived from all four cell types, including tDR‐37:74‐Glu‐CTC‐1‐M2 and tDR‐34:74‐Met‐CAT‐3 (Tables S3,S4, Supporting Information). Similar to the response of the cells to nutritional deprivation, these data suggest that the regulation of the biogenesis or stability of extracellular tDRs remains distinct from that of cellular tDRs in all the cell types examined. Furthermore, there appear to be several key “common” extracellular signatures associated with hypoxia in the rodent and human‐derived cell lines.

### 2.8 Oxidative Stress‐Shaped Cellular and Extracellular tDR Signatures

The generation of tDRs through tRNA cleavage has been shown to be a conserved response to oxidative stress in eukaryotes.^[^ ^45^ ^]^ Hence, we characterized the oxidative stress‐specific tDR signatures in the four cell types we utilized. Interestingly, most of the DE tDRs, including both cellular and extracellular tDRs, were downregulated upon H_2_O_2_ treatment in HEK and BeWo cells; in contrast, oxidative stress induced by ReO_2_ led to a significant increase of extracellular tDRs in both CM and CF cells. Cellular miRNAs or extracellular miRNAs only demonstrated minor changes during oxidative stress response (Figure S14A–H, Supporting Information).

954 cellular tDRs and 2301 extracellular tDRs were identified as significantly changed in HEK cells in response to H_2_O_2_ treatment (Table S5, Supporting Information). We also confirmed by northern blots, the top DE upregulated (cellular tDR‐39:72‐Asp‐GTC‐2‐M2) and downregulated (extracellular tDR‐1:34‐Pro‐CGG‐1‐M2) tDRs (Figure S15A–D, Supporting Information). 574 DE cellular tDRs and 1153 DE extracellular tDRs were observed in BeWo cells after H_2_O_2_ treatment (Table S5, Supporting Information). The 107 cellular tDRs that were significantly changed in both HEK and BeWo cells were generated from a variety of tRNA isotypes, including tRNA‐Leu, tRNA‐Arg, tRNA‐Tyr, and tRNA‐Lys (Figure S15E, Supporting Information). In the extracellular samples, all of the 695 DE extracellular tDRs that were altered by H_2_O_2_ treatment in both HEK and BeWo cells were significantly downregulated, and over 50% of them were derived from tRNA‐Glu, tRNA‐Pro, and tRNA‐Ser (Figure S15E, Table S5, Supporting Information). The top four cellular tDRs and top eight extracellular tDRs that were modulated by H_2_O_2_ in both cell types are shown in Figure S15F, Supporting Information.

As detailed above, to better phenocopy the oxidative stress in models of cardiac ischemia/reperfusion, our model of oxidative stress for CMs and CFs involved exposure to OGSD (0.2% O_2_ in GSD condition) for 5 h and ReO_2_ for an additional 24 h. Unexpectedly, there were no DE tDRs for CF cells but there were 18 significantly changed tDRs in CM cells after ReO_2_ (Figure S15G, Table S6, Supporting Information). In contrast to the changes noted with the HEK and BeWo cells, 2164 and 1911 extracellular tDRs from CM and CF cells were dramatically altered after ReO_2_, with most of them being upregulated (Figure S15G, Table S6, Supporting Information), consistent with the tDR expression tracing plots (Figure S13E–H, Supporting Information). 1195 overlapping extracellular tDRs were significantly modulated by ReO_2_ in both CM and CF cells and about 70% of them are derived from tRNA‐Glu and tRNA‐Pro (Figure S15G, Table S6, Supporting Information). The top four downregulated extracellular tDRs and the top eight upregulated extracellular tDRs in both cell types after the treatment of ReO_2_ are enumerated in Figure S15H, Supporting Information. Of interest, we noticed decreased expression level of tDR‐1:36‐Glu‐CTC‐1 and the increased levels of tDR‐1:30‐Glu‐CTC‐1 and tDR‐2:31‐Glu‐CTC‐1 in both CM and CF Exs in response to ReO_2_ (Table S6, Supporting Information); the distinct regulation of these tDRs derived from the same parent tRNA is suggestive of different modes of biogenesis or differential stability of these fragments. Strikingly, we also uncovered several extracellular conserved tDRs among human and rat species that are downregulated by oxidative stress, including tDR‐42:74‐Ser‐GCT‐2, tDR‐42:74‐Arg‐CCT‐4, and tDR‐39:74‐Leu‐AAG‐2 (Tables S5,S6, Supporting Information). Our data strongly suggested that the cellular context is essential for interpreting the response to oxidative stress. In the case of all the cell lines, extracellular tDRs were far more altered than cellular tDRs in response to oxidative stress. While the stress models were different between the human and rodent‐derived cells, the differences in the directionality of the changes in the extracellular tDRs were notable.

### 2.9 Specific and Shared Extracellular tDR Signatures among Three Profiled Stressors

**Figure 4:**
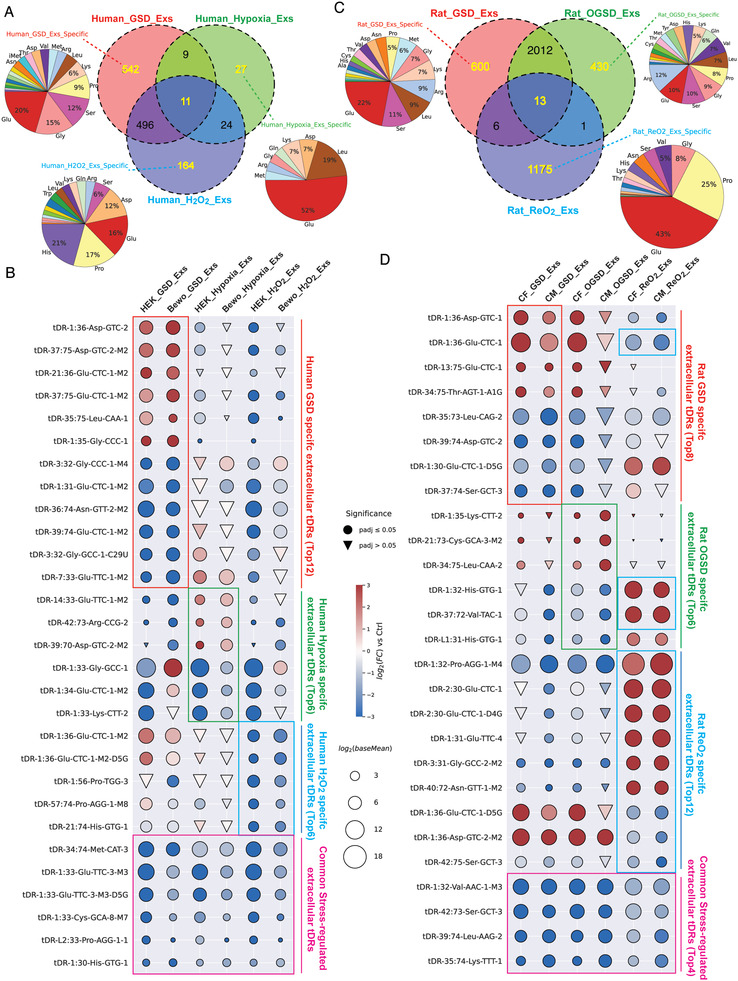
Specific and shared extracellular tDR signatures among three profiled stressors. A) Numbers of the specific and shared extracellular tDRs altered by three profiled stressors in HEK and BeWo cell‐derived Exs and their parent tRNA isotype distribution. B) The most significantly regulated extracellular tDRs that were specifically for GSD, hypoxia, and H_2_O_2_, and were shared among three stressors in HEK and BeWo cell‐derived Exs. C) Numbers of the specific and shared extracellular tDRs altered by three profiled stressors in CF and CM cell‐derived Exs and their parent tRNA isotype distribution. D) The most significantly regulated extracellular tDRs that were specifically for GSD, OGSD, and ReO_2_, and were shared among three stressors in CF and CM cell‐derived Exs.

It is well established that the levels of tDRs in human liquid biopsy can dynamically change under different diseases.^[^ ^24^ ^]^ However, knowledge of the primary variables influencing extracellular tDR levels remains unclear. To define the stressor‐specific and shared extracellular tDR signatures, an overlap of these DE extracellular tDRs identified under the three stressors in both HEK and BeWo cells was performed (**Figure** **4**A). Interestingly, about half of GSD‐altered extracellular tDRs (507 out of 1058) and half of the hypoxia‐shaped extracellular tDRs (35 out of 71) were also regulated by H_2_O_2_ (Figure 4A). 11 extracellular tDRs were downregulated in Exs by all three profiled stressors (Figure 4A). These results indicated that there are shared stress response mechanisms among nutritional deprivation, hypoxia, and oxidative stress. Notably, more than 50% of the 542 extracellular tDRs that were specifically regulated by GSD in both HEK and BeWo cells are derived from tRNA‐Glu, tRNA‐Gly, tRNA‐Ser, and tRNA‐Pro, while the majority of oxidative stress specially regulated extracellular tDRs are generated from tRNA‐His, tRNA‐Pro, tRNA‐Glu, and tRNA‐Asp (Figure 4A). There are only 27 hypoxia‐specific DE extracellular tDRs, and half of them are derived from tRNA‐Glu (Figure 4A). The top stress‐specific extracellular tDR transcripts and universal stress‐altered extracellular tDRs in human cell culture models are shown in Figure 4B.

The analysis of rodent stress‐shaped extracellular tDR signatures revealed more pronounced changes. Although the majority of GSD‐altered and OGSD‐regulated extracellular tDRs are overlapping, there are still 600 extracellular tDRs that were only altered by GSD and 430 extracellular tDRs that were only modulated by OGSD (Figure 4C). Interestingly, only a few DE extracellular tDRs from GSD and OGSD groups are also regulated by ReO_2_ treatment, indicating distinct stress responses in the three groups (Figure 4C). Unlike the GSD‐specific and OGSD‐specific extracellular tDRs that are derived from a variety of parent tRNAs, the identified 1175 extracellular tDRs that were specifically regulated by ReO_2_ are dominantly generated from tRNA‐Glu and tRNA‐Pro (Figure 4C). 12 out of the 13 extracellular tDRs significantly modulated by GSD, OGSD, and ReO_2_ treatments are downregulated. The top stress‐specific extracellular tDR transcripts and universal stress‐altered extracellular tDRs in rat cell culture models are shown in Figure 4D.

### 2.10 Patient Plasma tDR Signatures Reveal Distinct Stress Responses during Cardiac Surgery with CPB

**Figure 5:**
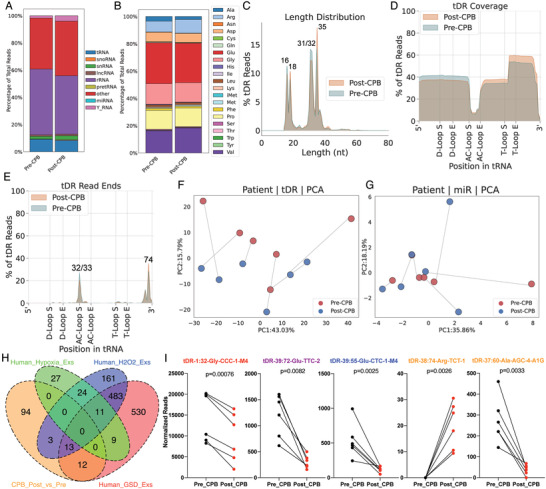
Patient plasma tDR signature reveals distinct stress responses during CPB surgery. A) About 10% total reads mapped to tRNA genes in the examined human plasma Exs samples. B) A large proportion of plasma tDRs are derived from tRNA‐Glu, tRNA‐Gly, tRNA‐Pro, and tRNA‐Val. C) Plasma tDRs are mainly 16–18 or 31–35 nts in length. D) Plasma tDRs are predominantly tRNA halves that derived from both ends of tRNA genes. E) Plasma tDRs end at position 32, 33, 72, or 74 of tRNAs. PCA analysis based on F) tDR landscapes provides better resolution to distinguish pre‐CPB from post‐CPB surgery than the one based on G) miRNA expression. H) CPB surgery‐modulated tDRs are overlapped with nutritional deprivation‐shaped and oxidative stress‐shaped extracellular tDR signatures but not hypoxia‐shaped extracellular tDR signature. I) Representative CPB surgery‐modulated tDRs that also found in GSD‐shaped extracellular tDR signatures (red), in GSD and H_2_O_2_‐regulated extracellular tDR signatures (purple), and in H_2_O_2_‐shaped extracellular tDR signatures (blue), and not found in three profiled stress‐specific extracellular tDR signatures (orange). Paired two‐tailed *t*‐test.

Cardiac surgery remains one of the most commonly performed major surgeries for patients with valvular abnormalities or multivessel coronary diseases.^[^ ^46^ ^]^ During the procedure, the heart is typically arrested and connected to a CPB machine, which provides both perfusion pressure and oxygenation to support the circulation.^[^ ^47^ ^]^ During this short time period, the heart is exposed to metabolic and oxidative stress.^[^ ^48^ ^]^ As a pilot “test” case to determine the applicability of our extracellular tDR signatures to human subjects, we collected the plasma samples from human patients at the initiation (Pre‐CPB) and about 73 min of CPB (Post‐CPB). ARM‐seq was performed on RNAs isolated from these plasma EV samples. The mapping results showed that around 10% of the total reads were mapped to tRNA genes in these human plasma samples (**Figure** **5**A); this detection was far more robust than previously reported for plasma using conventional small RNA‐seq in previous studies.^[^ ^27^, ^49^ ^]^

The distribution of reads from distinct tRNA isotypes reveals that a large proportion of tDRs in human plasma Exs are derived from tRNA‐Glu, tRNA‐Gly, or tRNA‐Pro (Figure 5B), similar to our findings from cell culture (Figure S2A, Supporting Information). More than 60% of plasma tDRs are 31–35 nts in length, and most of them are tRNA halves from either 5′ end or 3′ end of tRNA genes, which end at position 32 (anticodon loop) for the 5′ end, 72 (before the last nucleotide of 3′ end) or 74 (after the first nucleotide of 3′ CCA tail) (Figure 5C–E). These plasma tDR fragmentation profiles are consistent with the extracellular tDR fragmentation profile from cell culture (Figure 2). Although a proportion of tDRs, which were 16–18 nts in length and were generated by dual cleavages at T‐loop and position 72, were only found in plasma Exs but not in the Exs from our cell culture systems, our findings still indicates that extracellular tDRs present in the cell culture systems and plasma may share pathways of biogenesis.

PCA analysis based on tDR abundance profiles showed modest resolution for distinguishing pre‐CPB surgery patients from post‐CPB surgery patients based on tDR signatures, while the PCA analysis based on miRNA expression profiles showed limited resolution to distinguish the two populations (Figure 5F,G). This suggests that extracellular tDRs may have potential as markers of the stress response. Strikingly, we identified 122 significantly changed plasma tDRs following CPB, with 41 of them being upregulated (Figure 5H). Next, we selected tDRs that were significantly changed during CPB and assessed any overlap with our three common‐stress‐specific extracellular tDRs signatures. 25 out of 122 DE tDRs after 73 min of CPB surgery were found in the nutritional deprivation‐specific extracellular tDR signatures and 13 of them overlapped with both nutritional deprivation‐specific and oxidative stress‐specific extracellular tDR signatures; none of them were represented in the hypoxia‐specific extracellular tDR signatures (Figure 5H). tDR‐1:32‐Gly‐CCC‐1‐M4, tDR‐39:72‐Glu‐TTC‐2, and tDR‐39:55‐Glu‐CTC‐1‐M4 were significantly downregulated following CPB, in common with exposure to nutritional deprivation or oxidative stress in our cell culture systems (Figure 5I). Notably, among the other 94 DE plasma tDRs that did not overlap with our cell culture models, tDR‐38:74‐Arg‐TCT‐1 can only be detected in patients after CPB and tDR‐37:60‐Ala‐AGG‐4‐A1G dramatically decreased after completion of CPB (Figure 5I). Overall, these results support prior data suggesting that cells experience oxidative stress and nutritional deprivation during CPB and provide a novel circulating RNA signature for these cellular processes.

### 2.11 ANG and RNASE1 Critically Contribute to the Stress‐Modulated Cellular and Extracellular tDR Signatures

**Figure 6:**
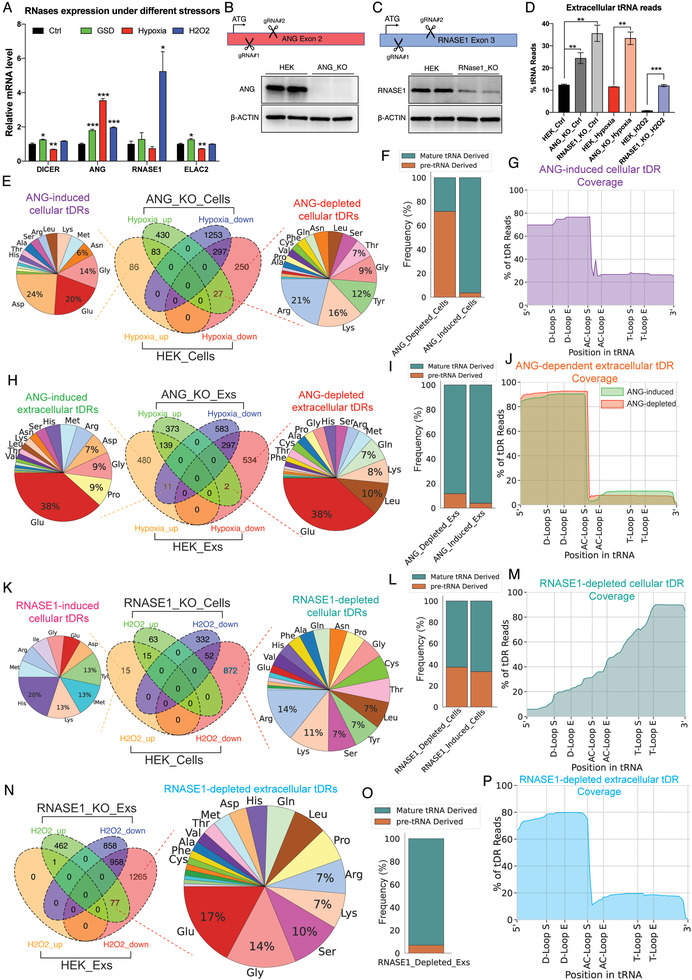
ANG and RNASE1 critically contribute to the stress‐modulated cellular and extracellular tDR signatures. A) The expression levels of four ribonucleases after the treatment of three different stressors in HEK cells. B) The strategy and western blot validation of ANG knockout in HEK cells using CRISPR/Cas9 with paired gRNAs. C) The strategy and western blot validation of RNASE1 knockout in HEK cells using CRISPR/Cas9 with paired gRNAs. D) Both ANG and RNASE1 knockout significantly increase the tRNA reads in exRNA samples. E) Numbers and parent tRNA isotype distribution of the ANG‐dependent cellular tDRs. F) ANG‐induced cellular tDRs are dominantly derived from mature tRNAs while ANG‐depleted cellular tDRs are mainly derived from pre‐tRNAs. G) ANG‐induced cellular tDRs are mainly tRNA halves. H) Numbers and parent tRNA isotype distribution of the ANG‐dependent extracellular tDRs. I) Both ANG‐induced and ANG‐depleted extracellular tDRs are mainly derived from mature tRNAs. J) ANG‐depleted extracellular tDRs are 1 nucleotide longer than ANG‐induced extracellular tDRs. K) Numbers and parent tRNA isotype distribution of the RNASE1‐dependent cellular tDRs. L) More than 60% of RNASE1‐depleted cellular tDRs are derived from mature tRNAs. M) Coverage plot of RNASE1‐depleted cellular tDRs. N) Numbers and parent tRNA isotype distribution of the RNASE1‐depleted extracellular tDRs. O) Most of the RNASE1‐depleted extracellular tDRs are derived from mature tRNAs. P) Coverage plot of RNASE1‐depleted extracellular tDRs.

Recent small RNA sequencing data have uncovered a vast array of tDRs; however, mechanisms dictating their biogenesis and expression still remain largely unclear. Present knowledge suggests that tDR production depends on several ribonucleases, including ANG, RNASE1, ELAC2, and DICER.^[^ ^20^ ^]^ However, their roles in the biogenesis of cellular and extracellular tDRs during stress response have rarely been studied. To further investigate the mechanisms of biogenesis and expression of cellular and extracellular tDRs, we first examined the expression levels of these ribonucleases in HEK cells subjected to different stressors. Interestingly, in hypoxia, ANG is most significantly increased, and RNASE1 is dramatically increased after H_2_O_2_ treatment (**Figure** **6**A). To explore their functional roles in tDR biogenesis in response to hypoxia and oxidative stress, respectively, ANG and RNASE1 were knocked out individually using CRISPR/Cas9 tools with paired gRNAs in HEK cells and the resulting monoclonal cell lines were selected for further study. Genomic DNA PCR results (Figure S16A,B, Supporting Information) and western blot results (Figure 6B,C) clearly demonstrated the successful and complete knockout of ANG and RNASE1. However, there was still a minor amount of RNASE1 protein in the RNASE1‐knockout cells, which could be attributed to either the presence of RNASE1 in cell culture medium or to the antibody cross reactivity with other RNases. Next, ANG‐knockout cells were exposed to hypoxia treatment, and RNASE1‐knockout cells were treated with H_2_O_2_ for 24 h. The cellular RNAs and exRNAs from these cells with or without treatments were then isolated and sequenced using ARM‐seq. Interestingly, both ANG and RNASE1 knockout significantly increased the overall extracellular tRNA/tDR reads at both baseline and stress conditions with minor influences on the total cellular tRNA/tDR reads (Figure 6D; Figure S16C, Supporting Information), suggesting ANG and RNASE1 may play critical roles in regulating the stability of subpopulations of extracellular tDRs by subjecting them to degradation; silencing of these RNAses would then be expected to increase the expression of these tDRs. The composition of tRNA isotypes within the exRNA samples were also changed after ANG or RNASE1 knockout, especially with regards to tRNA‐Gly, tRNA‐Pro, and tRNA‐Ser (Figure S16D, Supporting Information).

To identify the cellular and extracellular tDRs that are targeted by ANG in response to hypoxia, differential expression analysis was performed. In the ANG‐knockout HEK cells, 540 cellular tDRs were significantly increased, and 1550 cellular tDRs were significantly decreased after hypoxia treatment (Figure 6E). After overlapping with the hypoxia‐shaped cellular tDR signatures from HEK wild type cells, we identified 86 hypoxia‐upregulated tDRs whose expression levels were no longer significantly increased in ANG‐knockout cells (Figure 6E), suggesting these 86 cellular tDRs are induced by ANG in response to hypoxia. Notably, these 86 ANG‐induced cellular tDRs were mainly derived from mature tRNAs, including tRNA‐Asp, tRNA‐Glu, and tRNA‐Gly, and most of them are tRNA halves with lengths of 32/33, 36, and 41 nts, and the cleavage sites at position 33 or 36 (Figure 6E–G; Figure S16E,F, Supporting Information). These results are consistent with previous studies that demonstrate ANG is actively involved in cellular tDR biogenesis through the cleavage of the anticodon loop of a subset of tRNAs.^[^ ^50^ ^]^ In addition, 277 cellular tDRs that were significantly downregulated during hypoxia in wild type cells were not found to be significantly altered under hypoxic conditions in ANG‐knockout cells (Figure 6E), which suggests that they are targeted by ANG for degradation during hypoxic response. Unexpectedly, these 277 ANG‐depleted cellular tDRs are mainly derived from tRNA precursors (pre‐tRNAs), including tRNA‐Arg, tRNA‐Lys, tRNA‐Tyr, and tRNA‐Gly (Figure 6E,F), which indicates that ANG could be involved in tRNA maturation. The top four ANG‐induced, ANG‐depleted, and ANG‐independent cellular tDRs in the setting of hypoxic stress are shown in Figure S16G, Supporting Information.

Similarly, we uncovered 491 ANG‐induced extracellular tDRs whose expression levels are upregulated during the hypoxic response in wild‐type cells but not ANG‐knockout cells and 536 ANG‐depleted extracellular tDRs that are only downregulated in wild type HEK cells after hypoxia treatment (Figure 6H). They are both primarily derived from mature tRNAs, with a similar tRNA isodecoder composition (Figure 6H,I). Strikingly, most of the ANG‐depleted extracellular tDRs are 33 nts long with cleavage at position 33, which is one nucleotide longer than the ANG‐induced extracellular tDRs (Figure 6H–J; Figure S16H,I, Supporting Information). For example, upon hypoxia treatment, extracellular tDR‐1:33‐Glu‐CTC‐1‐M2‐D5G is significantly decreased and extracellular tDR‐1:32‐Glu‐CTC‐1‐M2‐D5G is dramatically increased, while they both remain constant when ANG is knocked out (Figure S16J, Supporting Information). These results suggest that the presence of ANG may be necessary to transform the 33 nts tDRs to generate the 32 nts tDRs in the extracellular environment.

Distinct from ANG, RNASE1 is rarely involved in tDR biogenesis but predominantly contributes to targeting tDRs for degradation in response to H_2_O_2_ treatment (Figure 6K). Only 15 RNASE1‐induced cellular tDRs were identified, while 872 RNASE1‐depleted cellular tDRs were observed (Figure 6K). These RNASE1‐depleted cellular tDRs are derived from various tRNA molecules, including both mature tRNAs and pre‐tRNAs, with a wide range of lengths and cleavage sites (Figure 6K–M; Figure S16K–M, Supporting Information). Similarly, no RNASE1‐induced extracellular tDR is found, whereas 1342 extracellular tDRs are degraded by RNASE1 during H_2_O_2_ treatment (Figure 6N). These RNASE1‐depleted extracellular tDRs are mainly tRNA halves with 31/32 nts long and are derived from mature tRNAs (Figure 6N–P; Figure S16N–P, Supporting Information), which is the main population of extracellular tDRs in the HEK‐derived Exs at baseline (Figure 3A,E,I). We also noticed that the overall extracellular tRNA reads are dramatically decreased from 12% to 0.8% after H_2_O_2_ treatment but are restored to a normal level through RNASE1 knockout (Figure 6D). Together, these findings suggest that RNASE1 is targeting a large population of cellular and extracellular tDRs for degradation under oxidative stress.

### 2.12 Identification of AGO2‐Dependent Extracellular tDRs

**Figure 7:**
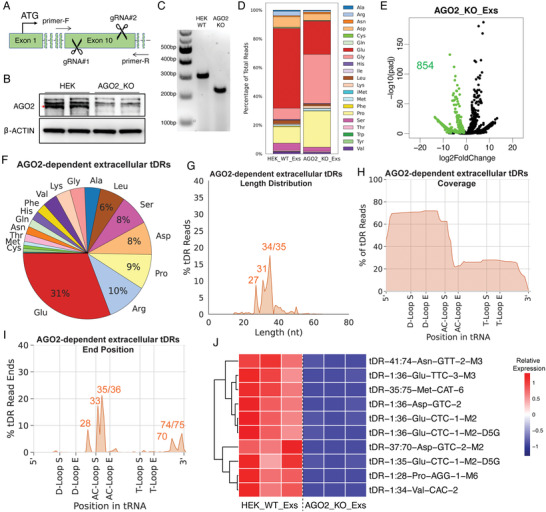
Identification of the AGO2‐dependent extracellular tDRs. A) The Strategy of AGO2 knockout in HEK cells using CRISPR/Cas9 with paired gRNAs. B) Western blot validation of AGO2 knockout cells. C) Genomic DNA PCR validation of AGO2 knockout cells. D) The tRNA isotype types of the Exs derived from wild type or AGO2‐knockout cells. E) Volcano plot identified 854 extracellular tDRs that were significantly downregulated after AGO2 knockout as AGO2‐dependent extracellular tDRs. F) The parent tRNA isotype distribution of the AGO2‐dependent extracellular tDRs. G) The length distribution of AGO2‐dependent extracellular tDRs. H) Coverage plot of AGO2‐dependent extracellular tDRs. I) The end positions of AGO2‐dependent extracellular tDRs. J) Heatmap shows the top ten most significant AGO2‐dependent extracellular tDRs.

Emerging evidence has shown that exRNAs include abundant, full‐length tRNAs and tDRs while the underlying mechanism by which these tRNAs/tDRs are released to the extracellular environment remains poorly understood.^[^ ^25^ ^]^ Of note, AGO2 has been shown to extensively export miRNAs from cells to extracellular environments as either free AGO2 complex or embedded in EVs.^[^ ^51^ ^]^ To elucidate whether AGO2 is involved in the transportation of extracellular tDRs, we built AGO2‐knockout HEK monoclonal cell lines using CRISPR/Cas9 (**Figure** **7**A). After validating the successful knockout of AGO2 using genotyping and western blot (Figure 7B,C), we collected their conditional medium together with those from wild‐type HEK cells and sequenced the Exs using ARM‐seq. The extracellular tRNA composition differs between AGO2‐knockout and wild‐type cells, especially the tRNA‐Glu, tRNA‐Gly, and tRNA‐Pro (Figure 7D). Differential expression analysis identified 854 extracellular tDRs that are significantly decreased upon AGO2 knockout (Figure 7E), suggesting the transportation or stability of these extracellular tDRs depend on AGO2. These AGO2‐dependent extracellular tDRs are mainly tRNA halves derived from tRNA‐Glu, tRNA‐Arg, tRNA‐Pro, tRNA‐Asp, and tRNA‐Ser (Figure 7F–H). Notably, these AGO2‐dependent extracellular tDRs have a wide length distribution with peaks at 27, 31, 34, and 35 nts and various termination sites at positions 28, 33, 35, 36, 70, 74, and 75, on tRNA molecules (Figure 7G–J), while the main population of extracellular tDRs in the HEK‐derived Exs at baseline are 31–33 nts long and end at positions 33 and 74 (Figure 3A,E,I). For instance, tDR‐1:36‐Glu‐TTC and tDR‐1:36‐Glu‐CTC are AGO2‐dependent, whereas the expression levels of their 33nt forms, including tDR‐1:33‐Glu‐TTC and tDR‐1:33‐Glu‐CTC, did not change after AGO2 knockout (Figure 7J, Table S7, Supporting Information). These results suggest that AGO2 is necessary for transporting or stabilizing a sub‐population of extracellular tDRs.

## 3 Discussion

The expanding use of RNA sequencing technology has led to an explosion in the discovery of novel RNA species. Recently, considerable advances have been made with regards to our understanding of the non‐coding transcriptome and its derivatives. tDRs, which were initially described in bacteria as the 3D structure of tRNA was being solved, hinted at non‐canonical functions through interaction with other cellular systems.^[^ ^52^, ^53^ ^]^ A range of subsequent studies demonstrated that these fragments were generated through a regimented process and implicated several ribonucleases important in their biogenesis.^[^ ^16^, ^20^, ^45^ ^]^ tRNA fragments are classified based on their position relative to the parental molecule and include 5′‐tRNA halves or fragments, 3′‐tRNA halves or fragments, and internal fragments; together, we refer to them as tDRs. Notably, several studies have suggested key functional roles for tDRs in the cellular response to stress, affecting fundamental processes such as mRNA stability, silencing, ribosomal function, and stress granule formation.^[^ ^16^, ^20^ ^]^ Finally, tDRs appear to constitute a significant proportion of RNAs found in the Exs across different biofluids, and their export from cells has been shown to affect cellular phenotypes or mediate intercellular signaling.^[^ ^54^ ^]^ However, the presence of modifications on tRNA bases (that may prevent consistent reverse transcription), and the rapidly evolving algorithms for mapping and naming tDRs has been an obstacle in generating a comprehensive atlas of cellular and extracellular tDRs in response to different cellular stressors.

We have previously described a methodology that leverages the activity of enzymes that demethylate commonly modified bases in tRNA to better identify previously unreported tDRs.^[^ ^19^ ^]^ Here we systematically use ARM‐seq to comprehensively profile the cellular and extracellular tDR landscape in four different cell types under different cellular perturbations. Our study provides for the first time, a comprehensive atlas of tDR signatures for cellular stress, and surprisingly demonstrates the dynamic changes in extracellular tDRs in response to stress. In a small pilot study, we demonstrate the possible application of these signatures to human studies; in patients undergoing CPB, a model of oxidative stress and nutritional deprivation, we note the dynamic changes in key tDRs previously identified in our cellular studies. More importantly, we explore the functional roles of ANG and RNASE1 in the biogenesis and stability regulation of both cellular and extracellular tDRs by leveraging ARM‐seq and CRISPR/Cas9 technologies. Finally, we identify a specific sub‐population of extracellular tDRs which are transported or stabilized by AGO2. In addition to providing a comprehensive tDR signature for different cellular stressors, our data provide an initial mechanistic understanding of the roles of RNases and RNA binding proteins in the expression of extracellular tDRs.

### 3.1 ARM‐seq to Identify Cellular and Extracellular tDRs

As expected from our prior work, ARM‐seq led to a dramatic improvement in the detection of tDRs, with a significant increase in both the proportion of reads mapping to tRNA genes, as well as longer reads that spanned known methylation sites. Most notable was the robust detection of key tDRs in response to cellular stress that were not detected by conventional small RNA sequencing (Figure S1G,H, Supporting Information). Therefore, this study represents a far more accurate and comprehensive atlas of the tDR signatures of cellular stress.

This study represents the first use of ARM‐seq to detect the profile of extracellular tDRs in response to cellular stress. We chose to examine these profiles across a diverse set of cell lines from two different species (rodent and human) to define whether there are “common” signatures to different cellular stressors. At the same time, we found interesting differences in the proportion of tDRs (both cellular and extracellular) in the cells. For example, CMs appear to have higher tDR expression at baseline and both CMs and CFs show a robust up‐regulation of extracellular tDRs upon ReO_2_. Whether different tissues have significant variance in the overall tDR expression at baseline (in the absence of stress) is unclear; furthermore, any functional implication of this finding warrants future investigation. A general theme that emerged when examining the tDRs across the different cells was that the extracellular profile of tDRs in terms of abundance and type diverged significantly from the cellular tDRs. For example, higher proportions of tDRs derived from tRNA‐Glu and tRNA‐Pro were observed in the Exs. Our findings complement other published studies,^[^ ^29^, ^30^ ^]^ notably the presence of 5′ halves from tRNA‐Glu‐TTC, tRNA‐Glu‐CTC, tRNA‐Gly‐GCC, and tRNA‐Gly‐CCC. However, the use of ARM‐seq has allowed for the detection of species other than 5′‐halves that have been previously described in the aforementioned studies, such as the 3′‐halves derived from tRNA‐Asp‐GTC, tRNA‐Glu‐CTC, and tRNA‐Asn‐GTT, 3′ fragments derived from tRNA‐Ser‐GCT and tRNA‐Pro‐AGG, and those without conventional names, especially those tDRs derived from pre‐tRNAs. Like the previously mentioned studies, our work also confirmed the preponderance of 5′‐tRNA halves ending in the anticodon loop, but also demonstrated the presence of 3′‐halves and intracellular tDRs that have not been previously recognized in the Exs. Interestingly, the extracellular tDRs derived from the 3′ halves had distinct ends (at position 74) compared to their cellular counterparts. Importantly, we complement and extend our ARM‐seq results with northern blots, validating the dynamic changes of key ARM‐seq‐identified stress‐regulated cellular and extracellular tDRs in HEK cells treated with three different stressors. While the sheer number of DE tDRs across the different cell types and stressors preclude the possibility of validating every single DE tDR in all the profiled samples, the northern blotting data we have are a sufficient sample size to corroborate the reliability of ARM‐seq data to identify DE tDRs in response to stress.

### 3.2 Extracellular tDRs as Unique Signatures of Cellular Stress

Our results demonstrated that extracellular tDRs had distinct non‐overlapping signatures in response to the different stressors and improved discrimination between the different stressors compared to miRNAs. Interestingly while cellular tDRs changed with stress, as has been previously demonstrated, the pattern of expression changes showed considerable more overlap between the different stressors than the extracellular tDRs. This pattern was seen in all four cell types examined, suggesting that extracellular tDRs may provide better discrimination in measuring cellular stress than extracellular miRNAs or cellular tDRs. We systematically tracked cellular and extracellular tDRs that were altered in response to each of the stressors (nutrient deprivation, hypoxia, or oxidative stress) in each cell line to determine if there were signatures that were common to all cell lines, and if there were tDRs that were unique to individual cell types. When examining nutrient deprivation in the human cell lines, we found far more extracellular tDRs (1058 total) that were regulated in the same direction in both HEK and BeWo cells. The top upregulated tDRs were derived from the 5′ halves of tRNA‐Gly (which has been previously shown to be a significant contributor to EV‐contained tDRs^[^ ^30^ ^]^). In contrast, the top down‐regulated tDRs were derived from tRNA‐Glu. Similar to the human cell lines, a large number of extracellular tDRs were altered in the rodent CM and CF cells. Importantly, we found a set of extracellular tDRs (Figure S11, Supporting Information) that were commonly upregulated (≈46 tDRs) or down‐regulated (≈97 tDRs) in both species. If validated in multiple other cell types, these may serve as a “universal marker” for nutrient deprivation or metabolic stress.

Similar findings were noted with hypoxic stress, although it appeared that most cellular and extracellular tDRs were decreased with hypoxic stress. Interestingly, the cancer BeWo cell line showed less pronounced changes than HEK cells in this regard. Whether cancer cells are more resistant to hypoxia would be of interest to address in the future. Compared to these cell lines, the cardiac primary rodent cells had a far higher number of altered extracellular tDRs, with a number of these being common between all the cell types. The generation of tDRs in response to oxidative stress has been shown to be a conserved response in eukaryotic cells. Interestingly, we found some marked differences between the human cell lines (most cellular and extracellular tDRs were decreased) and the primary cardiac cells (where ReO_2_ increased most extracellular tDRs). These data suggested that the cellular context was influential in the biogenesis, export, or stability of extracellular tDRs in response to oxidative stress. Together our findings suggest that extracellular tDRs have distinct signatures and sequences that do not simply reflect the stoichiometry of cellular tDRs.

### 3.3 Extracellular tDRs as Biomarkers for Human Diseases

The potential of tDRs to serve as biomarkers to diagnose disease or monitor disease progression has been studied to some extent in cancer patients.^[^ ^55^ ^]^ However, our findings that extracellular tDRs do not necessarily mirror the stoichiometry of cellular tDRs should caution investigators about extrapolating altered levels of tDRs in tissues (such as cancer cells) and expecting similar changes in plasma. Our finding that extracellular tDRs are readily altered with cellular stress led us to query whether similar changes may happen in human subjects and whether some of the signatures we observed in our cell models were translatable to a clinical context. We examined the plasma tDRs in a pilot study of patients undergoing CPB, a model of oxidative stress and nutrient deprivation in human subjects. By measuring tDRs at the initiation (pre‐stress) and average 73 min after CPB (peak stress), we could compare the levels of individual tDRs. Interestingly, we found that similar to our cellular experiments, we detected both 5′ and 3′ tRNA halves in plasma with ARM‐seq and that the cleavage pattern was similar to what was noted in the extracellular tDRs in the cell culture system. We again found that plasma tDRs could modestly discriminate between pre‐stress and peak‐stress samples while miRNAs failed to do so. Interestingly, 25 out of 122 DE tDRs after average 73 min of CPB were common with our cellular models of nutrient deprivation, while 16 of these overlapped with signatures of oxidative stress. One of them, tDR‐38:74‐Arg‐TCT‐1, could only be detected 73 min after CPB.

It should be noted that this was a preliminary pilot study with a small sample size; however, we were encouraged by our results that some of the tDR signatures of cellular stress we have identified could be readily translated into a clinical context. Whether the presence of these signatures or degree of change is associated with clinical outcomes would be of paramount importance in future studies.

### 3.4 New Insights of the Biogenesis, Stability Regulation, and Transportation of tDRs

Notwithstanding emerging studies focusing on the functions of tDRs in different pathophysiological processes, the biogenesis of these tDRs, especially the extracellular tDRs, still remains unclear. We leveraged the technologies of ARM‐seq and CRISPR/Cas9 to study the roles of ANG and RNASE1 in tDR biogenesis in response to hypoxia and oxidative stress, respectively, since ANG is most significantly induced upon hypoxia and RNASE1 is dramatically activated by oxidative stress in HEK cells. Consistent with previous studies, we found that ANG mainly induces cleavage of the anticodon loop of tRNA‐Asp, tRNA‐Glu, and tRNA‐Gly to generate tRNA halves in response to hypoxia. Strikingly, we also, for the first time, demonstrated that ANG is involved in the stability regulation of a population of cellular tDRs that derived from pre‐tRNAs. The function of ANG in the biogenesis of extracellular tDRs has not been reported previously. We demonstrated that ANG is involved in the biogenesis of 491 extracellular tDRs and the stability of 536 extracellular tDRs. Interestingly, ANG‐depleted extracellular tDRs are one nucleotide longer than ANG‐induced extracellular tDRs, and some of them share the same parent tRNAs, such as tRNA‐Glu‐CTC, which highlights the possibility that ANG may be implicated in trimming some of ANG‐depleted tDRs to generate the ANG‐induced tDRs. Another evidence of ANG‐mediated tDR degradation is the overall extracellular tRNA reads from ANG knockout groups are significantly higher than wild‐type groups.

Unlike ANG, RNASE1 is primarily involved in the stability regulation of cellular and extracellular tDRs as only 15 RNASE1‐induced cellular tDRs were identified. RNASE1 knockout increased the overall extracellular tRNA reads under oxidative stress. Furthermore, we identified 872 cellular tDRs and 1352 extracellular tDRs potentially targeted by RNASE1 for degradation in response to oxidative stress. RNASE1‐depleted cellular tDRs are derived from a wide range of tRNAs, including both mature and pre‐tRNAs, indicating that RNASE1 has a wide range of cellular targets. The RNASE1‐depleted extracellular tDRs also have broad origins, and these are distinct from ANG‐depleted extracellular tDRs, which suggests that the RNases secreted from cells have unique specificity in targeting extracellular tDRs for degradation in response to different stressors. Our data are consistent with other studies that suggests that the stability (from degradation) may be important in the expression of extracellular tDRs.^[^ ^26^, ^56^ ^]^ However, we additionally demonstrate the active roles of ANG in the biogenesis of both cellular and extracellular tDRs and provide detailed characterization of the populations of tDRs for which ANG and RNASE1 are critically involved in the stability control in response to cellular stress.

As a key partner associated with extracellular miRNAs, AGO2 has been extensively reported to export miRNAs from cells and protect miRNAs from degradation in circulation.^[^ ^51^ ^]^ tDRs are small RNAs with sizes reminiscent of miRNAs, which raised the possibility that tDR exportation/protection is AGO2‐dependent. To address this question, we generated AGO2‐knockout cells and profiled their extracellular tDR expression. Although 854 AGO2‐dependent extracellular tDRs are identified, these do not constitute the primary population of extracellular tDRs with the read lengths of 31–33 nts, demonstrating AGO2 only associates with a subset of extracellular tDRs. These results may also be in line with previous studies suggesting that cellular tDRs mainly associate with AGO1, AGO3, and AGO4, but not AGO2.^[^ ^57^ ^]^ Of note, our results suggest that AGO2 may be responsible for either the transportation of these AGO2‐dependent extracellular tDRs from cells or protection of them from RNase‐mediated degradation thereby increasing their stability and relative expression levels.

### 3.5 Limitations

We recognize that we did not make a concerted effort to separate different exRNA carriers (e.g., riboproteins, EVs, of different size or density) in these studies, as this would have been technically difficult to conduct ARM‐seq on different carriers due to sample size constraints. As previously noted, the tDRs we describe may be associated both with EVs or with non‐EV carriers as has been previously shown.^[^ ^58^ ^]^ While several studies have reported that a significant majority of extracellular tDRs are carried in association with riboproteins, other studies have demonstrated the presence of tDRs within EVs,^[^ ^30^ ^]^ and importantly, a functional role of EV‐contained tDRs.^[^ ^59^ ^]^ Future studies would be needed to explicitly study the export and packaging of extracellular tDRs. Additionally, there has been recognition that exogenous tDRs or RNases from bovine serum used for cell culture may confound analysis of extracellular tDRs; while we have used bovine serum depleted of EVs by ultracentrifugation, we cannot exclude the presence of these confounders. Our RNASE1 knockout experiments confirm that endogenous RNASE1 plays an important role in the stability of ex‐tDRs in response to stress. Additionally, for most of our stressors, the same medium was also used for the stress conditions, making it unlikely that the changes we observed in tDRs could be attributed to such confounders. For those experiments that necessitated the use of different media for the stress conditions (such as GSD or OGSD), it should be noted that serum deprivation in these models leads to both up‐regulation and down‐regulation of key tDRs, arguing against exogenous RNases (contained in serum) being the sole factor in generation of our tDR signatures. Nonetheless, it remains a possibility that exogenous RNases may be a confounder for a subset of the extracellular tDRs noted.

Furthermore, we did not explore the functional implications of these findings. Whether extrusion of tDRs in response to particular stressors leads to changes in cellular phenotypes as has been shown for T‐cell activation,^[^ ^60^ ^]^ or whether cellular stress leads to an upregulation of extracellular tDR biogenesis would of great interest to determine in future studies. Whether these findings reflect distinct biogenesis, selective export, or differential stability for extracellular tDRs cannot be directly inferred from our study. It remains possible that the abundance of certain tDRs may reflect not selective biogenesis but increased stability of these fragments in the Exs compared to others,^[^ ^61^ ^]^ and support the findings of others that the biogenesis of extracellular tDRs may be distinct from cellular tDRs.^[^ ^24^, ^25^, ^32^ ^]^ These prior studies have demonstrated the export of full‐length tRNAs into the extracellular non‐EV fraction and subsequent processing by RNases to form tRNA halves.^[^ ^26^, ^32^ ^]^ ARM‐seq methodology was not designed to detect full‐length tRNAs, and it is therefore not surprising that we did not detect the parental tRNAs for the abundant tDRs that were detected. Other technologies such as DM‐tRNA‐seq together with the use of northern blot may have the ability to tackle this question. In terms of the translation of using extracellular tDRs as biomarkers for human diseases, stem‐loop‐based RT‐qPCR technology has been widely used to detect tDR expression, and the results are consistent with the high‐throughput sequencing data. However, this method is yet not possible to distinguish tDRs with only a few nucleotides difference. Emerging technologies are being developed to simplify the detection of the tRNA/tDR expression levels, including YAMAT‐seq,^[^ ^62^ ^]^ QuantM‐tRNAseq,^[^ ^63^ ^]^ mim‐tRNAseq,^[^ ^64^ ^]^ and Nanopore sequencing,^[^ ^65^ ^]^ which may ultimately lead to the clinical translation of these findings.

## 4 Conclusion

Using ARM‐seq, we have provided a comprehensive profile of cellular and extracellular tDRs in response to various cellular stressors in several different cell types. Our studies demonstrate that the profile of extracellular tDRs and their biogenesis may differ from cellular tDRs and that the response to stress leads to robust changes in the extracellular tDRs. Notably, extracellular tDRs provide far better discrimination between different types of cellular stressors than miRNAs. In a pilot study, we demonstrate the applicability of our findings in a human subject model of nutrient deprivation/oxidative stress. We explored the functional roles of stress‐induced RNases, ANG and RNASE1, in the biogenesis and stability regulation of cellular and extracellular tDRs and identified a sub‐population of AGO2‐transported/protected extracellular tDRs. We expect that our data may be of use to investigators in this field who wish to investigate extracellular tDRs as biomarkers for disease processes or examine their functional role in the context of cellular stress.

## 5 Experimental Section

### Generation of ANG, RNASE1, and AGO2 knockout HEK Cells

The knockout of ANG, RNASE1, and AGO2 was performed using CRISPR/Cas9 tools with paired gRNAs as previously described.^[^ ^66^ ^]^ Briefly, paired gRNAs targeting ANG exon2, RNASE1 exon3, and AGO2 exon10 were cloned into eSpCas9‐2A‐puro and eSpCas9‐2A‐GFP vectors (GenScript) respectively. HEK cells were transfected with paired gRNAs and selected with puromycin for 6 days. Serial diluted HEK cells were then seeded into 96 well plates and the resulted monoclonal cell lines were screened by genotyping and validated using western blots. The complete knockout monoclonal cell lines were then chosen for further experiments. gRNA sequences are listed in Table S8, Supporting Information.

### Cellular Small RNA and Extracellular RNA Isolation

Cells were immediately lysed by adding TRIzol (Thermo Fisher, Cat# A33251) after treatment and total RNAs were then isolated by following the manual. 40 µg total RNAs were subjected to small RNA isolation by using mirVana miRNA Isolation Kit (Thermo Fisher, Cat# AM1560). The cell culture medium was collected immediately after treatment and spun twice at 2000 g for 10 min to remove cell debris, followed by filtering through 0.8 µm filter. Next, 10–12 mL cell culture medium was subjected to extracellular RNA isolation using the exoRNeasy Maxi kit (Qiagen, Cat# 77164) with minor modifications. 20 µg mL^−1^ Glycogen was added into the upper aqueous phase before adding 2 volumes of 100% ethanol and the aqueous phase/ethanol mixture was incubated at −20 ℃ overnight to facilitate with small RNA precipitation. The exRNAs from 1 mL CPB patient plasma samples were isolated using the exoRNeasy Midi kit (Qiagen, Cat# 77044).

### Northern Blotting

Northern blotting was performed by following previous report,^[^ ^67^ ^]^ with significant modifications. Briefly, denatured 10–15 µg cellular total RNAs or 200–500 ng extracellular total RNAs were separated by 15% Criterion TBE‐Urea PreCast Gels (Bio‐Rad). The gels were stained with SYBR‐Gold (ThermoFisher Scientific) and transferred onto positively charged Nylon membrane (Sigma Aldrich) using Trans‐Blot Turbo Transfer System (Bio‐Rad). The membranes were then crosslinked with EDC at 60 ℃ for 1–2 h and prehybridized with ULTRAhyb Ultrasensitive Hybridization Buffer (Ambion). 50 pmol mL^−1^ Biotin‐labeled Locked Nucleic Acid‐modified DNA probes (designed and synthesized by Qiagen) were used for hybridization at 37 ℃ overnight. After washing sequentially with high stringent buffer, low stringent buffer, and 1× SSC, the blots were then processed and developed using Chemiluminescent Nucleic Acid Detection Module Kit (ThermoFisher Scientific).

### RNA Sample Pre‐Treatment and Small RNA Library Preparation for ARM‐Seq

Cellular small RNAs and exRNAs were successively treated with DNase I (Zymo Research, Cat# R1014), T4 Polynucleotide Kinase (New England Biolab, Cat# M0201L), and His‐AlkB. After each treatment, the RNA was cleaned to remove enzyme and buffer components using the RNA Clean & Concentrator‐5 kit (Zymo Research, Cat# R1014) before the next treatment, with the following minor modifications. 20 µg mL^−1^ Glycogen was added into sample/RNA Binding Buffer mixture before adding an equal volume of 100% ethanol and then the mixture incubated at −20 ℃ overnight to facilitate small RNA precipitation. Due to the presence of high dose heparin (250 units kg^−1^) in CPB patients undergoing cardiac surgery, the CPB patient plasma RNAs were pretreated with Heparinase I (New England Biolab, Cat# P0735L) at 30 ℃ for 3 h before DNase I treatment. Then, 100–500 ng His‐AlkB treated RNAs were used as input into the NEBNext Multiplex Small RNA Library construction kit (New England Biolab, Cat# E7300S) and instructions were followed with the exception of 60 ℃ temperature used for reverse transcription step. Size selection was performed by isolating the bands from 140 to 240 bp to remove the contamination of primer adaptors (around 127 bp) and longer RNAs. The quality of libraries was confirmed by High Sensitivity DNA ScreenTape Analysis (TapeStation, Agilent) and qPCR. Multiplexed libraries were sequenced on an Illumina HiSeq 2000 system with paired‐end 75 bp sequencing. About seven million read pairs were generated from each library.

### Mapping and tDR Naming

The accession numbers for the raw sequence data of in vitro stress response platforms reported in this paper are GEO: GSE173806 and GSE196072. Paired‐end sequencing reads were trimmed to remove their adapter sequences and merged using SeqPrep with the following parameters: ‐L 15 ‐A AGATCGGAAGAGCACACGTC ‐B GATCGTCGGACTGTAGAACTC. Reads were aligned to the pre‐built tRAX [http://trna.ucsc.edu/tRAX/] hg38 or rn6 reference database using Bowtie2 in very‐sensitive mode with the following parameters to allow for a maximum of 100 alignments per read: ‐‐very‐sensitive ‐‐ignore‐quals ‐‐np 5 ‐k 100. Utilizing the tRAX pipeline [https://github.com/UCSC‐LoweLab/tRAX], ENSEMBL (release 96) small ncRNA annotations were used to count the number of small RNA transcripts using only primary alignments to prevent double counting. For tDRs, tDRnamer [http://trna.ucsc.edu/tDRnamer/] was used to generate unique identifiers for each of the transcripts based on the isodecoder(s) they mapped to and positions of any misincorporations. The naming conventions for tDRnamer can be found at http://trna.ucsc.edu/tDRnamer/docs/naming/, and are based on the full‐length tRNA names derived from the Genomic tRNA Database.^[^ ^68^ ^]^ Raw counts were normalized using the DESeq2 package and utilized for downstream analyses.

### Read Length Distribution

To determine the read length distribution for different small RNA types reads were extracted that either aligned to a tRNA feature or another small RNA feature deemed “other.” The proportion of read lengths was then calculated by taking the number of reads corresponding to each length and dividing that by the total number of reads for that category (tRNA or other).

### tDR Abundance and End Distribution

To determine the abundance distribution for tDRs across a consensus tRNA transcript, tDR normalized read counts were extracted. For each of these transcripts, tRNA mapping information was used to determine which positions in the mature tRNA transcript these tDRs were derived from. Using this information, all tRNAs were collapsed into a “consensus” tRNA and the proportion of total abundance at each position was calculated by dividing the total abundance at each position in the consensus tRNA by the total number of tDR reads.

To determine the read end distribution for these tDRs across a consensus tRNA transcript, abundance values were attributed to only the final position the tDR mapped within the mature tRNA transcript. The end frequency was then calculated by dividing the total abundance at each position in the consensus tRNA by the total number of tDR reads.

### Dimensionality Reduction and Correlations

Using DESeq2 normalized values, dimensionality reduction was performed to assess the reproducibility of biological replicates. These analyses were performed for all tDRs and miRNAs, as annotated by ENSEMBL (release 96) by first extracting read counts for those transcripts prior to dimensionality reduction. PCA was performed using the Python package sklearn v0.23.2 and the first two components were used to generate scatter plots. UMAP projections for each of the small RNA types were generated using the Python package umap‐learn v0.5.0. Spearman correlation coefficients were calculated using the pandas v1.2.1 “corr” function with parameters: method = “spearman.”

### Tracking Plots

Tracking plots were generated to track the abundance of specific tDRs and miRNAs across different cellular conditions and/or environments using DESeq2 normalized values. Initially, tDRs were filtered to remove potential sequencing artifacts and degradation products by setting a cutoff threshold across all replicates. For miRNAs, all transcripts with >5 reads were retained. Biological replicates were merged and log2 values were calculated for all transcript types.

### Statistical Analysis

Statistical analyses of qPCR and patient sequencing data were performed using GraphPad Prism software (version 9). qPCR data are expressed as mean ± SEM and the statistical significance was assessed by two‐tailed unpaired Student's *t*‐test; for CPB patient ARM‐seq data, two‐tailed paired Student's *t*‐test was used; for all other ARM‐seq data, differential expression analysis was performed using DESeq2 to generate Benjamini–Hochberg‐corrected *p* values (*p*adj) to assess the statistical significance. The criterion for statistical significance was *p* < 0.05 (*p < 0.05, **p < 0.01, and ***p < 0.001).

### Animal and Human Statement

All animal studies were approved by the Massachusetts General Hospital Animal Care and Use Committee and under the guidelines on the use and care of laboratory animals for biomedical research published by National Institutes of Health (No. 85‐23, revised 1996). All human patient related studies were approved by the Partners Healthcare Institutional Review Board (Boston, MA) and included in the clinical trial NCT00985049 (https://clinicaltrials.gov/ct2/show/study/NCT00985049).

## Conflict of Interest

The authors declare no conflict of interest.

## Supporting information

Supporting Information

Supplemental Table 1

Supplemental Table 2

Supplemental Table 3

Supplemental Table 4

Supplemental Table 5

Supplemental Table 6

Supplemental Table 7

Supplemental Table 8

## Data Availability

The data that support the findings of this study are openly available in NIH_GEO at https://www.ncbi.nlm.nih.gov/geo/query/acc.cgi?acc=GSE173806, reference number 173806.
