# Supplementary material for: Distinct Stress‐Dependent Signatures of Cellular and Extracellular tRNA‐Derived Small RNAs
Source: Adv Sci (Weinh). 2022 Apr 4;9(17):2200829. doi: 10.1002/advs.202200829 (PMC9189662; doi:10.1002/advs.202200829)
Supplement: Supplementary file 1 — Supporting Information [file ADVS-9-2200829-s005.pdf]

ADVANCED SCIENCE
Open Access

Supporting Information

for *Adv. Sci.*, DOI 10.1002/advs.202200829

Distinct Stress-Dependent Signatures of Cellular and Extracellular tRNA-Derived Small RNAs

*Guoping Li, Aidan C. Manning, Alex Bagi, Xinyu Yang, Priyanka Gokulnath, Michail Spanos, Jonathan Howard, Patricia P. Chan, Thadryan Sweeney, Robert Kitchen, Haobo Li, Brice D. Laurent, Sary F. Aranki, Maria I. Kontaridis, Louise C. Laurent, Kendall Van Keuren-Jensen, Jochen Muehlschlegel, Todd M. Lowe and Saumya Das**

SUPPLEMENTAL INFORMATION

Distinct stress-dependent signatures of cellular and extracellular tRNA-derived small RNAs (tDRs)

Guoping Li, Aidan C. Manning, Alex Bagi, Xinyu Yang, Priyanka Gokulnath, Michail Spanos, Jonathan Howard, Patricia P. Chan, Thadryan Sweeney, Robert Kitchen, Haobo Li, Brice D. Laurent, Sary F. Aranki, Maria I. Kontaridis, Louise C. Laurent, Kendall Van Keuren-Jensen, Jochen Muehlschlegel, Todd M. Lowe, and Saumya Das.

SUPPLEMENTAL METHODS

Recombinant His-AlkB Expression and Purification

The expression and purification of recombinant His-AlkB were performed as previously described with modifications [1]. Briefly, the His-AlkB-AVA421 plasmid was transformed into *E. coli* BL21(DE3)pLysS competent cells (Promega, Cat# L1191) and cultured in Overnight Express™ Instant TB Medium (Sigma, Cat# 71491) at 37 ℃ to OD^{600} 1.2. The cell pellet was resuspended and lysed by sonication and the cell debris was removed by centrifugation. Then, the clarified supernatant from the crude extract was carefully collected and mixed with TALON Metal Affinity Resin (Takara Bio, Cat# 635502) for 1 hour to allow for the binding of His-tagged AlkB protein to resin. After several washing steps, the resin-bind proteins were eluted and separated by Econo-Column® Chromatography Columns (BioRad, Cat# 7371512). The protein concentration of eight collected fractions was measured and the three protein-enriched fractions were pooled anddialyzed successively in Dialysis Buffer and Storage Buffer. The dialyzed protein supernatant containing recombinant His-AlkB protein was validated by Coomassie Bule staining and westernblotting, and used for ARM-seq.

Human Cell Line Culture and Stress Treatment

HEK293 cells were purchased from ATCC (ATCC, CRL-1573) and cultured in D10 medium, which consisted of DMEM with high glucose and pyruvate (Thermo Fisher, Cat# 11995073) supplemented with 10% fetal bovine serum (FBS) (Thermo Fisher, Cat# 10437028) and 1% Penicillin/Streptomycin (Thermo Fisher, Cat# 15140122). BeWo cells (ATCC, CCL-98), were cultured in F-12K Medium (ATCC, Cat# 30-2004) containing 10% FBS and 1% Penicillin/Streptomycin. For the glucose and serum deprivation (GSD) treatment, HEK293 and BeWo cells were cultured in glucose-free DMEM basal medium for 24 hours. For the hypoxia treatment, HEK293 and BeWo cells were fed with complete medium and maintained in hypoxia chamber with 0.1% oxygen for 24 hours. For the oxidative stress treatment, HEK293 and BeWo cells were treated with 0.8 mM hydrogen peroxide (H_2O_2) and 1.7 mM H_2O_2 separately in complete medium for 24 hours. FBS used for control, hypoxia and H_2O_2 treatments was filtered through 0.2 μm filter and followed by 24 h ultracentrifugation at 120,000 g to deplete extracellularvesicles (EVs) before use.

Neonatal Rat Ventricular Cardiomyocytes (CMs) and Cardiac Fibroblast (CFs) Isolation and Stress Treatment

All animal procedures conformed to the animal welfare regulations of the Massachusetts General Hospital Subcommittee on Research Animal Care (SRAC). Neonatal rat ventricular CMs and CFs were isolated as previously described [2]. Briefly, the ventricular heart tissues were carefully dissected from 1-day old Sprague Dawley rat neonates and minced using a sterile sharp razor blade, followed by 8 rounds of digestion in the ADS buffer (NaCl 116 mM, HEPES 20 mM, NaH_2PO_4 1 mM, KCl 5.4 mM, $MgSO_4$ 0.8 mM, and Glucose 5.5 mM) containing 0.05 mg/ml collagenase type II (Worthington, Cat# LS004177) and 1 mg/ml pancreatin (Sigma, Cat# P3292). Then the single cell suspensions were pooled and plated onto 10 cm tissue culture dishes with CM medium [DMEM medium supplemented with 10% horse serum (Thermo fisher, Cat# 26050-088), 5% FBS and 1% Penicillin/Streptomycin] for 1 hour. The attached cells were considered to be CFs and maintained in D10 medium. The floating cells were then purified via Percoll (GE Healthcare, Cat# 17-0891-01) gradient; the cells in the middle layer, which were considered as CMs, were collected and maintained in CM medium.

For the GSD treatment, CF and CM cells were exposed to glucose-free DMEM basal medium for 5 hours. For the hypoxia treatment, CF and CM cells were fed with glucose free DMEM basal medium and maintained in hypoxia chamber with 0.2% oxygen for 5 hours. For the reoxygenation (ReO_2) treatment, CF and CM cells were exposed to hypoxia treatment for 5 hours and then fed with complete medium and cultured in normoxia for an additional 24 hours. FBS and horse serum used for control and ReO_2 treatment were also EV-depleted by filtration and ultracentrifugation.

CPB Patient Plasma Sample Collection

Plasma samples were prospectively collected from six patients undergoing elective aortic valve replacement surgery with cardiopulmonary bypass by a single surgeon at a single institution in 2020 (https://clinicaltrials.gov/ct2/show/NCT00985049). Written informed consent approved by the Partners Healthcare Institutional Review Board (Boston, MA) was provided. Baseline plasma samples were obtained from venous blood immediately after commencement of cardiopulmonary bypass, which included intermittent cold blood cardioplegia for myocardial protection. Post-ischemic samples were obtained immediately before removal of the aortic cross-clamp at the end of the de-airing procedure. Plasma samples were collected using K2EDTA tubes and stored at −80℃

before RNA isolation. Median ischemic time was 73 minutes (Inter-quartile range [IQR] 68-121). Of the six patients included in this analysis, 3 (50%) were female and the median age was 73 years (IQR 66-75).

Quantitative Polymerase Chain Reaction (qPCR)

qPCR was performed as previously described [3]. Briefly, 1 μg of cellular total RNA was reverse transcribed to cDNA using the High-Capacity cDNA Reverse Transcription Kit (Thermo Fisher, Cat# 4368813). qPCR was then performed using a QuantStudio 6 Flex Real-Time PCR Systems (Thermo Fisher) with SsoAdvanced™ Universal SYBR® Green Supermix (BioRad, Cat# 1725275). The sequences of qPCR primers used are listed in Table S8.

Western Blot

Western blotting was performed as previously described [3]. Briefly, cell pellets were resuspended in RIPA buffer (Thermo Fisher, Cat# 89900) containing Protease and Phosphatase Inhibitor Cocktail (Thermo Fisher, Cat# 78441), and PMSF, and lysed via sonication. The protein concentration of clarified cell lysate was then measured using the BCA protein assay kit (Thermo Fisher, Cat# 23227). 5 ~ 20 μg of total protein per lane was separated by SDS-PAGE gel and transferred to PVDF membrane. 5% BSA in TBST was used for blocking and primary antibody incubation. HRP conjugated secondary antibodies were then applied and the blots were developed using SuperSignal® West Femto Maximum Sensitivity Chemiluminescent Substrate (Thermo Fisher, Cat# 34094). The antibodies used were the following: HIF1a (Cell Signaling Technology, Cat# 14179S), DDIT3 (Cell Signaling Technology, Cat# 2895), DDIT4 (Proteintech, Cat# 10638-1-AP), r-H2AX (Cell Signaling Technology, Cat# 80312), LC3B (Cell Signaling Technology, Cat# 3868S), β-Actin (Sigma, Cat# A5316), ANG (R&D Systems, Cat# AF265), RNASE1 (ThermoFisher, Cat# PA5-28278), AGO2 (Abcam, Cat# ab57113), Goat Anti-Mouse Immunoglobulins/HRP (Dako, Cat# P0447), Goat Anti-Rabbit Immunoglobulins/HRP (Dako, Cat# P0448) , and Rabbit anti-Goat Immunoglobulins/HRP (Aglient, Cat#P044801-2).

Standardized tDR naming system

tDRnamer (http://trna.ucsc.edu/tDRnamer/) was used for the standardized tDR naming in this study, which consists of five parts as shown in the example below:

tDR-4:33-Val-AAC-1-M7-A10U

① ② ③ ④ ⑤

The instruction manual for tDRnamer can be found at http://trna.ucsc.edu/tDRnamer/docs/naming/. Briefly, ①. Prefix - "tDR" stands for "tRNA Derived RNA". ②. Position – This includes the start and end positions of the tDR relative to the source tRNA. If the position is located at the leader or trailer sequence of the precursor tRNA gene, the numbering will be preceded with a letter "L" or "T" respectively. ③. Source tRNA - This is the name of the tRNA from which the tDR is derived. tRNA names from Genomic tRNA Database are used. ④. Matching tRNA transcripts – If a tDR is mapped to multiple tRNA genes, an optional component with prefix "M" and the number of matching tRNAs will be added to the tDR name. ⑤. Variations – For a substitution, the annotation will include the base in tDR, the position of the substitution relative to the tDR, and the substituted base in the source tRNA. For an insertion, the annotation will include a prefix "I", followed by the position of the insertion relative to the tDR and the inserted base. For a deletion, the annotation will include a prefix "D", followed by the position before the deletion relative to the tDR and the deleted base from the source tRNA.

Reference:

1 Hrabeta-Robinson, E., Marcus, E., Cozen, A. E., Phizicky, E. M. & Lowe, T. M. High-Throughput Small RNA Sequencing Enhanced by AlkB-Facilitated RNA de-Methylation (ARM-Seq). *Methods Mol Biol* **1562**, 231-243, doi:10.1007/978-1-4939-6807-7_15 (2017).

2 Li, J. *et al.* Mir-30d Regulates Cardiac Remodeling by Intracellular and Paracrine Signaling. *Circ Res* **128**, e1-e23, doi:10.1161/CIRCRESAHA.120.317244 (2021).

3 Li, G. *et al.* Dysregulation of the SIRT1/OCT6 Axis Contributes to Environmental Stress-Induced Neural Induction Defects. *Stem Cell Reports* **8**, 1270-1286, doi:10.1016/j.stemcr.2017.03.017 (2017).

SUPPLEMENTAL FIGURES

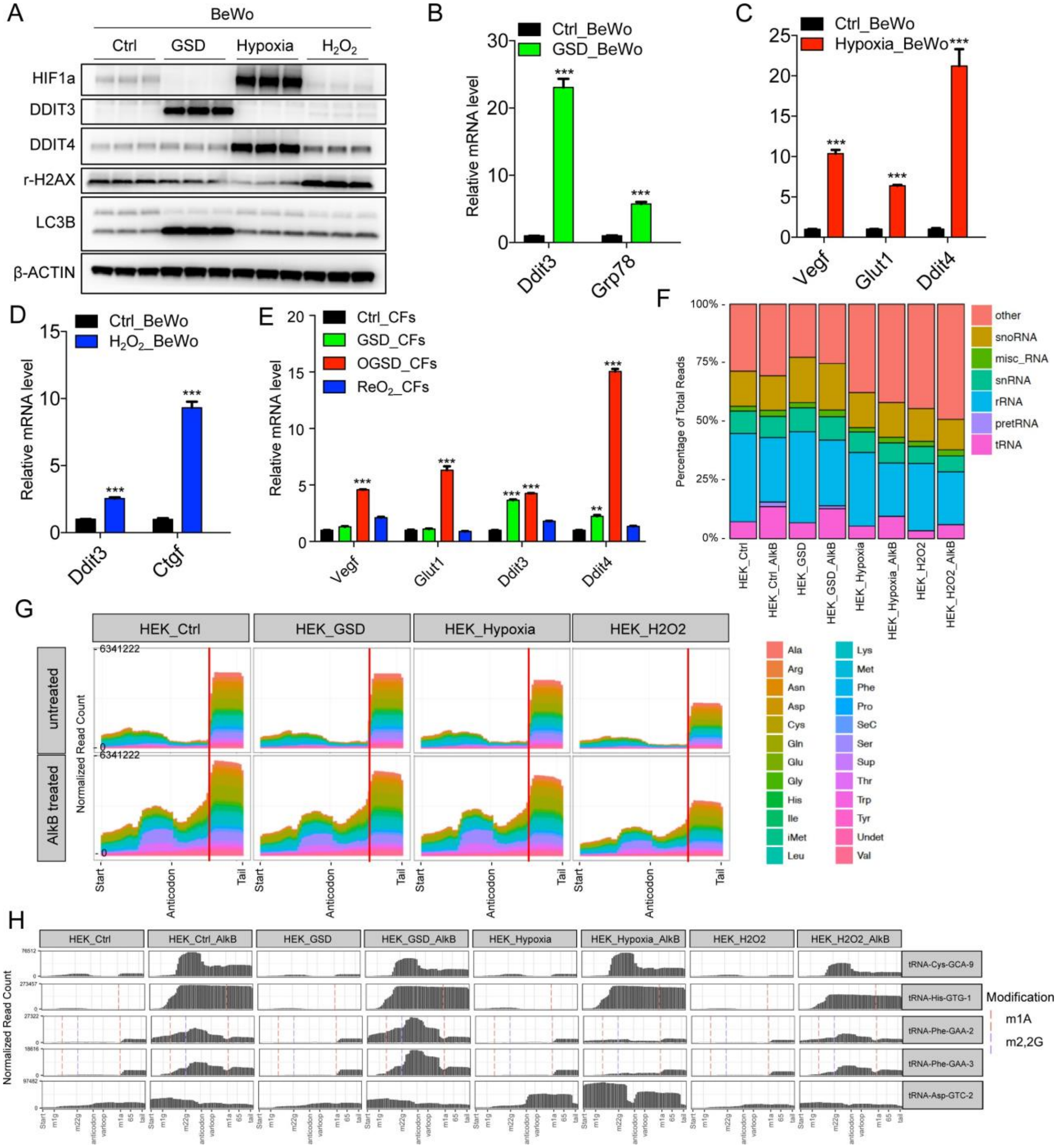

Figure S1. The validations of in vitro stress response platforms and the advantages of ARM- seq

A. Western blot validation of stress response in BeWo cells.

B. GSD induces Ddit3 and Grp78 expression in BeWo cells.

C. Hypoxia activates the expression of Ddit4 and angiogenesis-related genes, including Vegf and Glut1, in BeWo cells.

D. H2O2 treatment increases the expression levels of Ddit4 and Ctgf in BeWo cells.

E. QPCR validation of different stress responses in CFs: GSD activated the expression level of Ddit3 and Ddit4; OGSD induced the levels of Vegf and Glut1; ReO2 restored the levels of these genes induced by OGSD

F. AlkB treatment dramatically increases the proportion of tRNA reads.

G. AlkB treatment significantly elongates the read length of tRNA genes; red line indicates the m^1A modification at position 58.

H. tRNA coverage plots show four representative tRNAs that could only be detected by ARM-seq but not regular small RNA-seq.

Data are shown as means ± SEM of at least three independent experiments. The unpaired two-tailed Student's t test was used in (B) - (E). $^{**}p < 0.01$, $^{***}p < 0.001$ versus the control group.

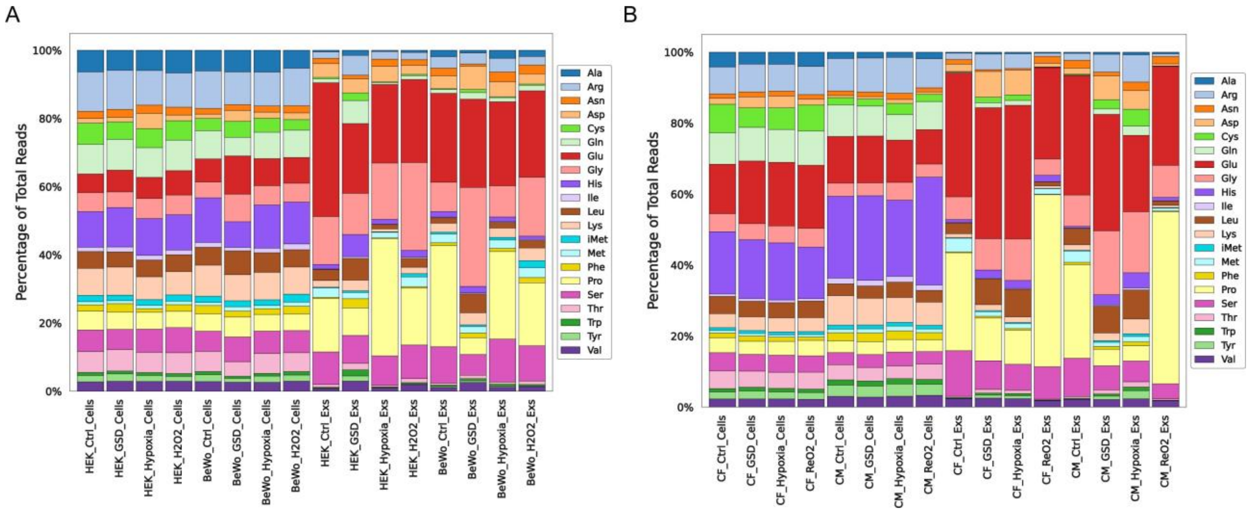

Figure S2. The tRNA isotype compositions of all the profiled samples.

Extracellular tDRs have isotype preferences from tRNA-Glu, tRNA-Gly and tRNA-Pro that are distinct from intracellular tDRs in both human (A) and rat (B) samples.

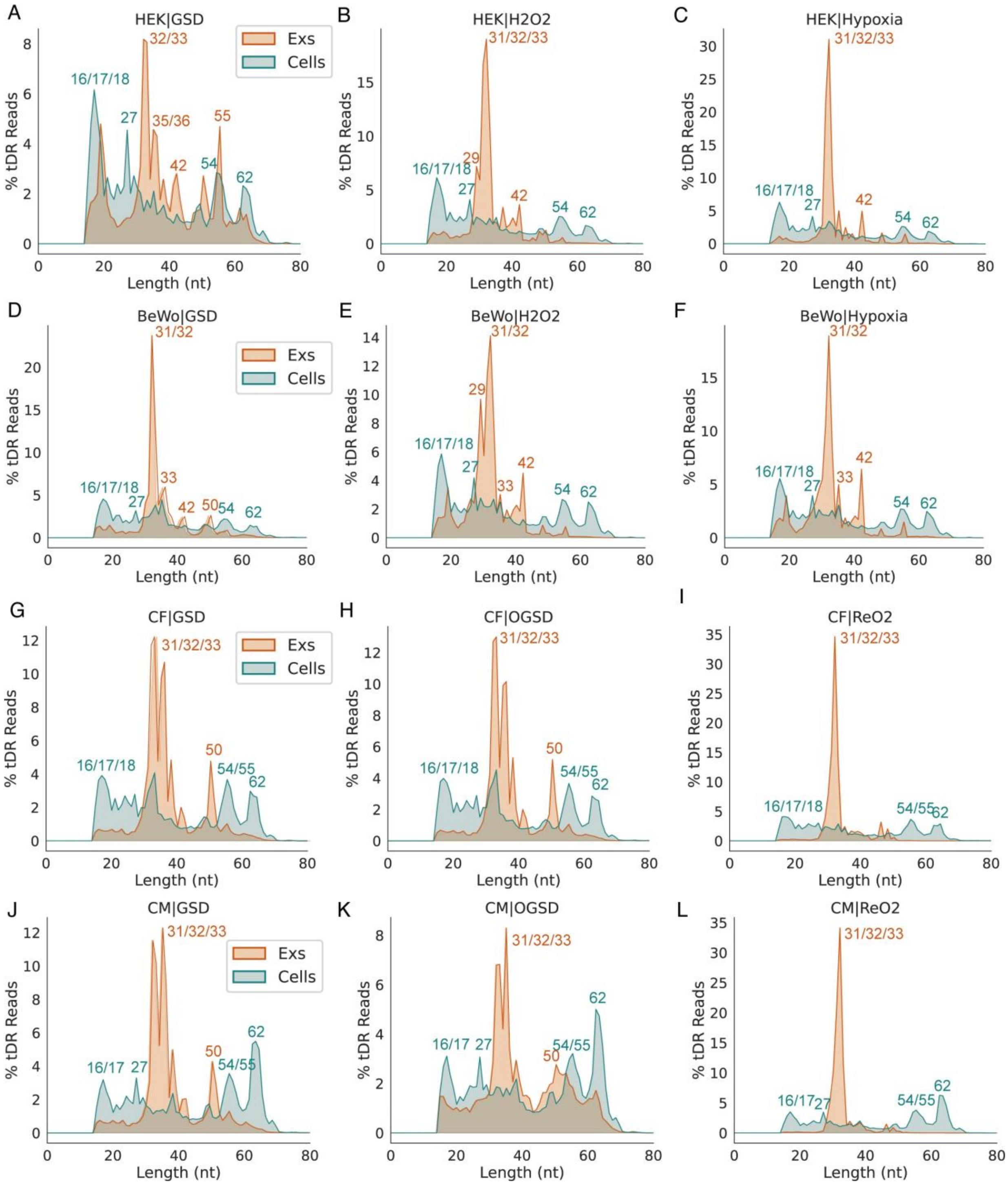

Figure S3. Extracellular tDRs are predominantly 31-35 nts length in both human and rat samples upon different stress treatments.

A-C. The length distribution plots of intracellular tDRs and extracellular tDRs during GSD (A), hypoxia (B) and H_2O_2 (C) treatment in HEK cells.

D-F. The length distribution plots of intracellular tDRs and extracellular tDRs during GSD (D),

hypoxia (E) and H_2O_2 (F) treatment in BeWo cells.

G-I. The length distribution plots of intracellular tDRs and extracellular tDRs during GSD (G), hypoxia (H) and ReO_2 (I) treatment in CF cells.

J-L. The length distribution plots of intracellular tDRs and extracellular tDRs during GSD (J), hypoxia (K) and ReO_2 (L) treatment in CM cells.

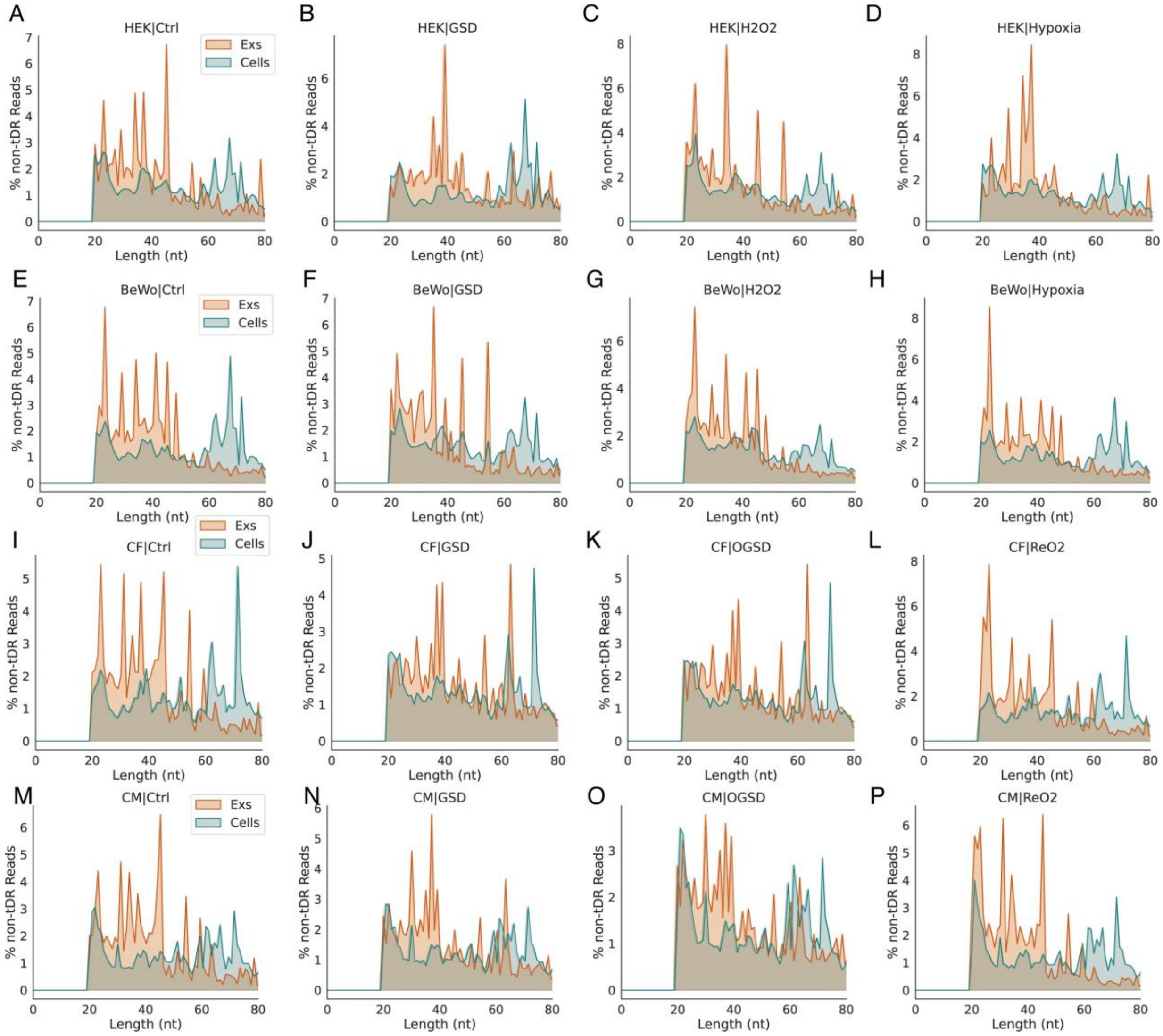

Figure S4. Other extracellular small RNA species do not have obvious length preference. A-D. The length distribution plots of intracellular and extracellular other small RNA species at baseline (A) and during GSD (B), hypoxia (C) and H_2O_2 (D) treatment in HEK cells. **E-H**. The length distribution plots of intracellular and extracellular other small RNA species at baseline (E) and during GSD (F), hypoxia (G) and H_2O_2 (H) treatment in BeWo cells. **I-L**. The length distribution plots of intracellular and extracellular other small RNA species at baseline (I) and during GSD (J), hypoxia (K) and ReO_2 (L) treatment in CF cells.**M-P**. The length distribution plots of intracellular and extracellular other small RNA species at baseline (M) and during GSD (N), hypoxia (O) and ReO_2 (P) treatment in CM cells.

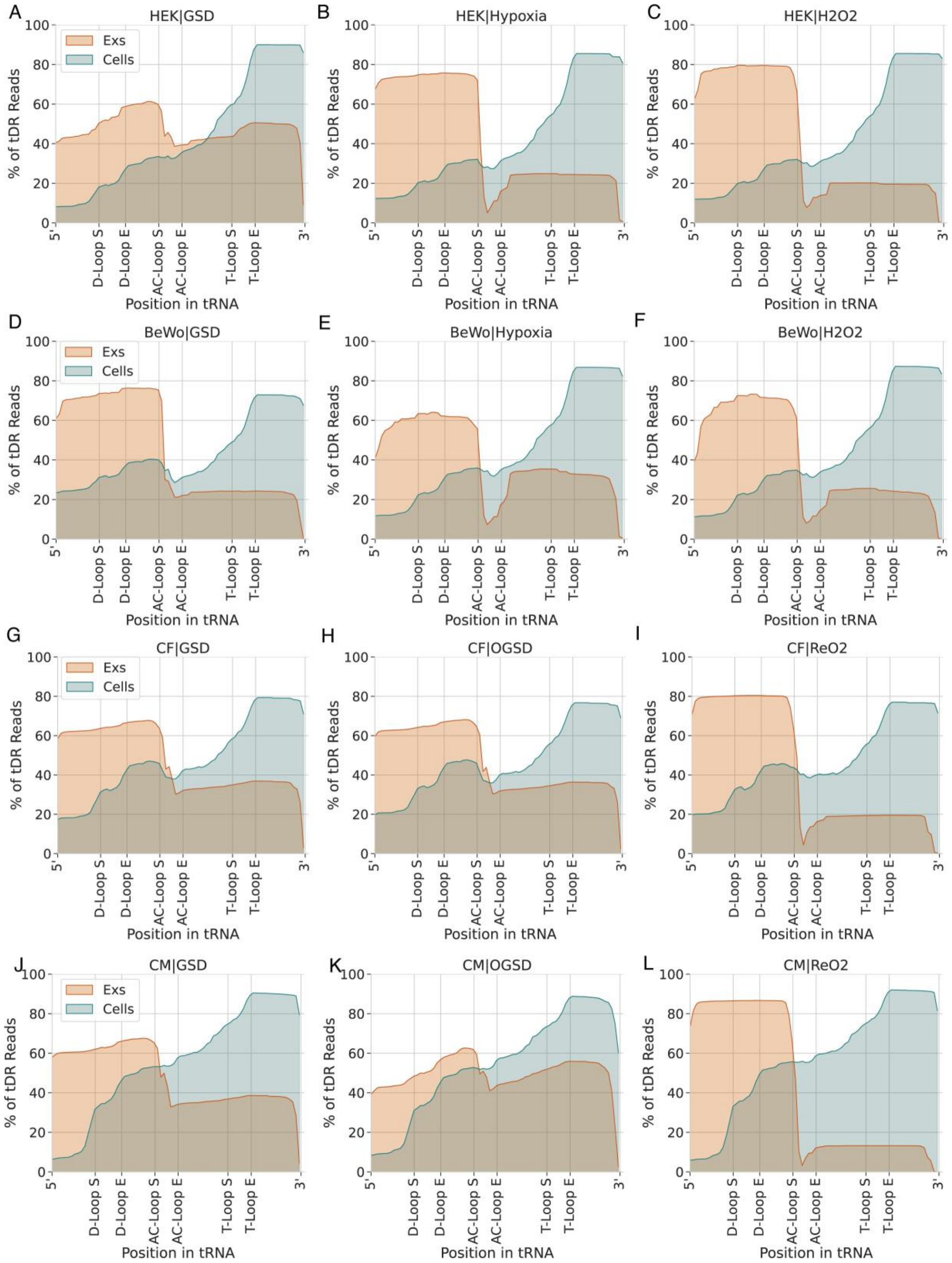

Figure S5. Extracellular tDRs are mainly tRNA halves while intracellular tDRs are predominantly derived from 3' end of tRNAs with wide range of length.

A-C. The coverage plots of intracellular tDRs and extracellular tDRs during GSD (A), hypoxia (B)

and H_2O_2 (C) treatment in HEK cells.

D-F. The coverage plots of intracellular tDRs and extracellular tDRs during GSD (D), hypoxia (E) and H_2O_2 (F) treatment in BeWo cells.

G-I. The coverage plots of intracellular tDRs and extracellular tDRs during GSD (G), hypoxia (H) and ReO_2 (I) treatment in CF cells.

J-L. The coverage plots of intracellular tDRs and extracellular tDRs during GSD (J), hypoxia (K) and ReO_2 (L) treatment in CM cells.

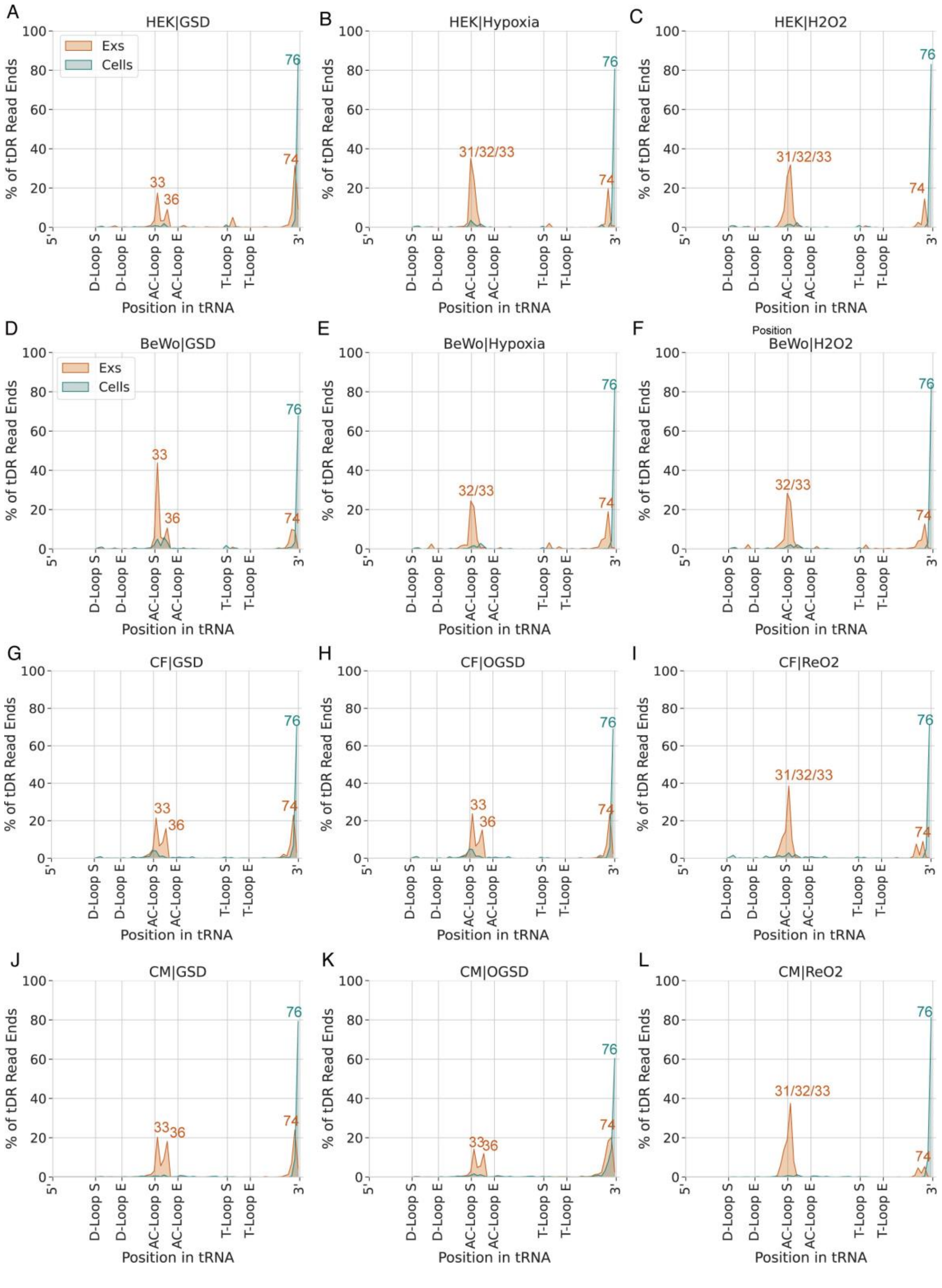

Figure S6. Extracellular tDRs mainly terminate at either the anticodon loop or position 74 of tRNA genes while intracellular tDRs predominantly terminate at position 76 of tRNA genes.

A-C. The end positions of intracellular tDRs and extracellular tDRs during GSD (A), hypoxia (B)

and H_2O_2 (C) treatment in HEK cells.

D-F. The end positions of intracellular tDRs and extracellular tDRs during GSD (D), hypoxia (E) and H_2O_2 (F) treatment in BeWo cells.

G-I. The end positions of intracellular tDRs and extracellular tDRs during GSD (G), hypoxia (H) and ReO_2 (I) treatment in CF cells.

J-L. The end positions of intracellular tDRs and extracellular tDRs during GSD(J), hypoxia (K) and ReO_2 (L) treatment in CM cells.

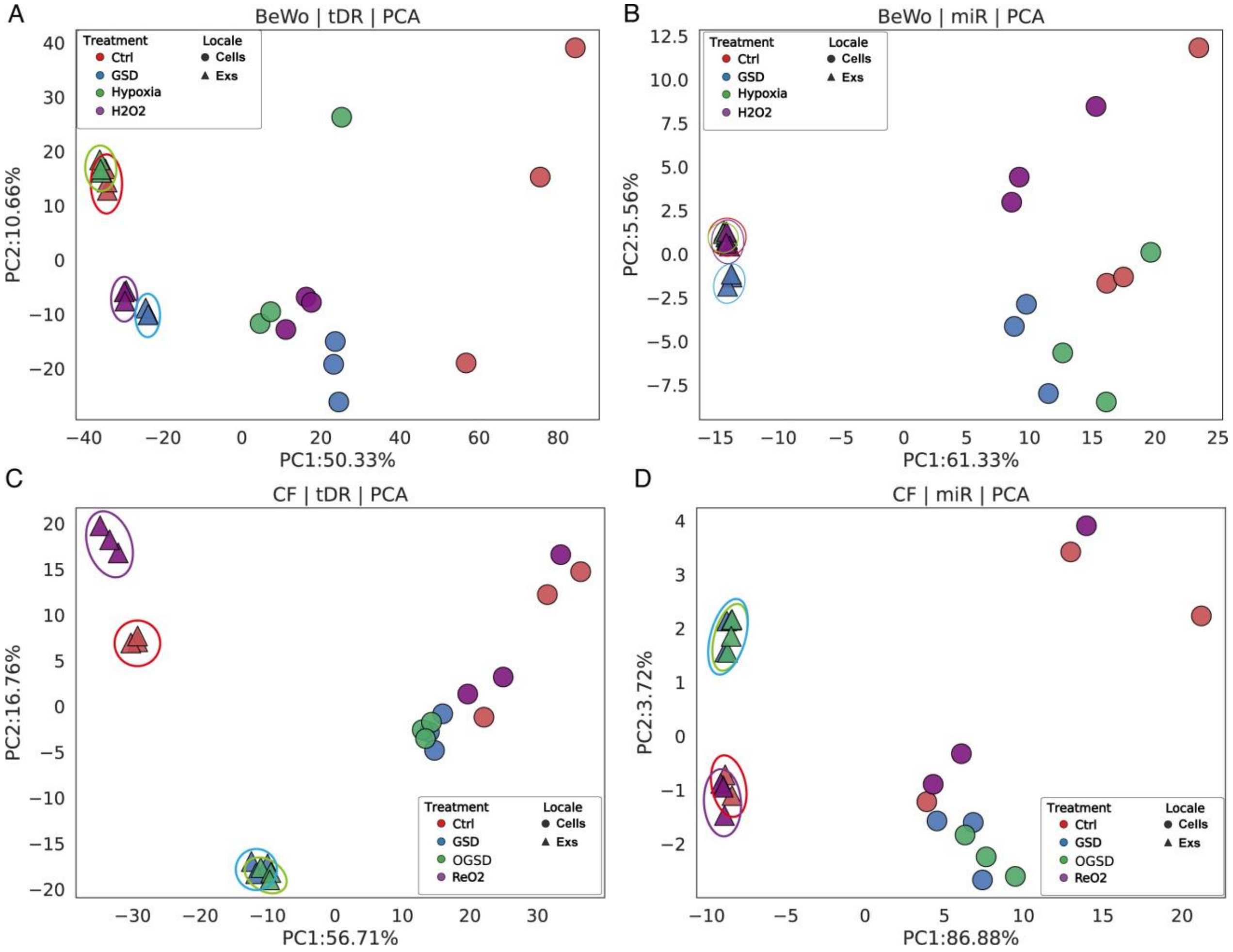

Figure S7. PCA analyses based on tDR profiles get better resolution to distinguish different stress responses than those based on miRNA expressions.

A and **B**. PCA analysis of expression values for tDR class (A) provides better resolution to distinguish the Exs samples (circled) derived from BeWo cells after different stress treatments than miRNA class (B).

C and **D**. PCA analysis of expression values for tDR class (C) provides better resolution to distinguish the Exs samples (circled) derived from CF cells after different stress treatments than miRNA class (D).

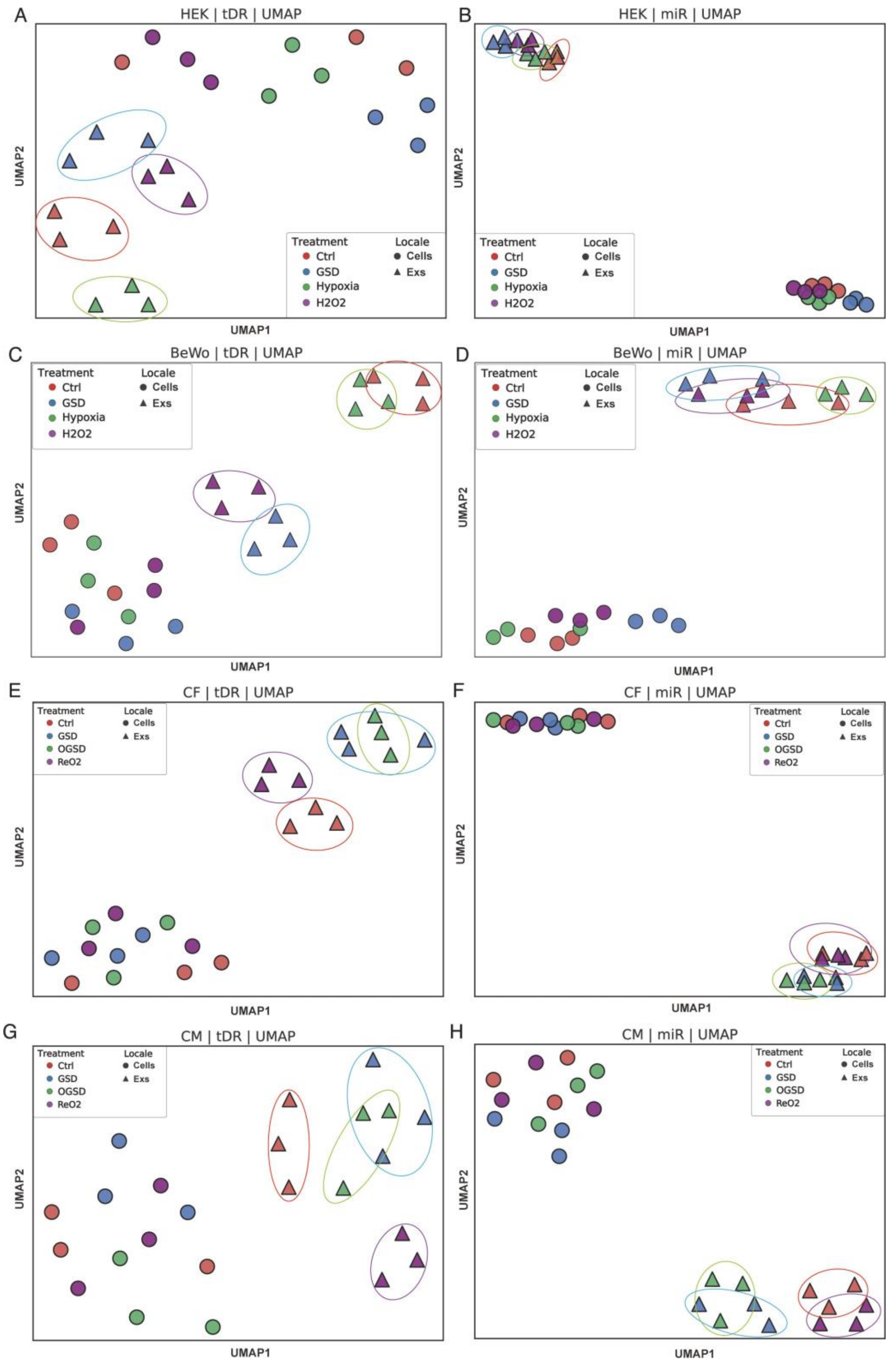

Figure S8. UMAP projection analyses show that extracellular tDR signatures provide better resolution to distinguish different stress responses than extracellular miRNA signatures

A and **B**. UMAP projection of expression values for tDR class (A) provides better resolution to distinguish different stress-specific Exs samples (circled) from HEK cells than miRNA class (B).
C and **D**. UMAP projection of expression values for tDR class (C) provides better resolution to distinguish different stress-specific Exs samples (circled) from BeWo cells than miRNA class (D).
E and **F**. UMAP projection of expression values for tDR class (E) provides better resolution to distinguish different stress-specific Exs samples (circled) from CF cells than miRNA class (F).
G and **H**. UMAP projection of expression values for tDR class (G) provides better resolution to distinguish different stress-specific Exs samples (circled) from CM cells than miRNA class (H).

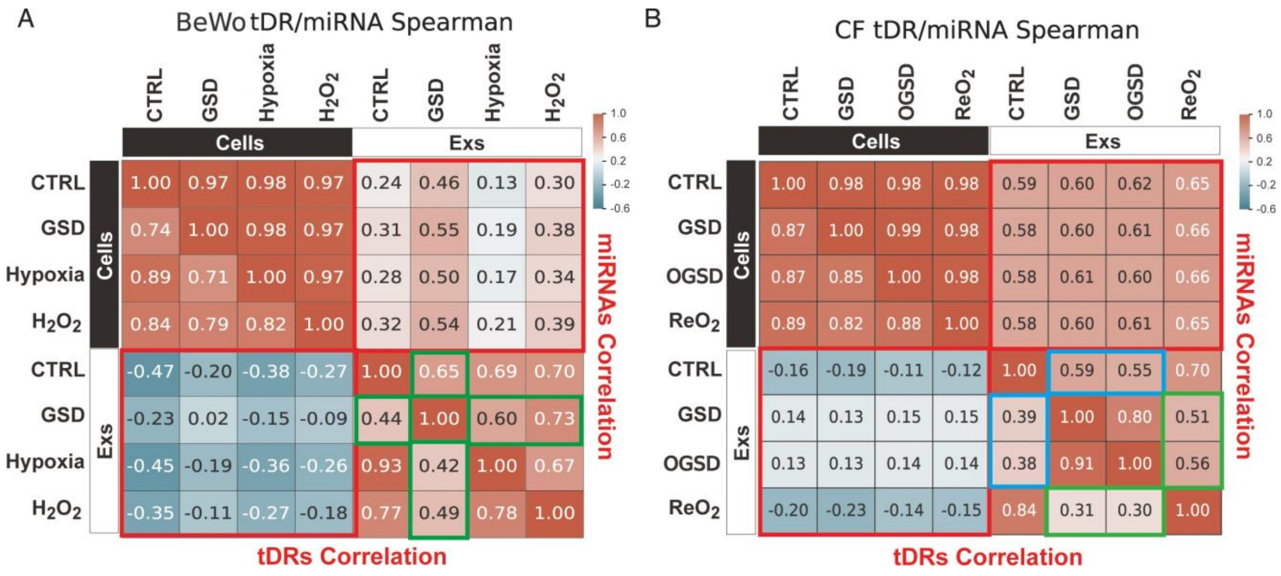

Figure S9. Extracellular tDR expression landscapes show larger variance among different stress-treated samples than extracellular miRNAs

A. Heatmaps of correlation coefficients (Spearman) for tDR class (left bottom) shows larger variance among different samples than miRNA class (right top) in BeWo cells.

B. Heatmaps of correlation coefficients (Spearman) for tDR class (left bottom) shows larger variance among different samples than miRNA class (right top) in CF cells.

Red boxes show the difference between intracellular samples and extracellular samples; blue boxes indicate the difference of extracellular samples between each stressor and control group; green boxes show the difference between different stressors.

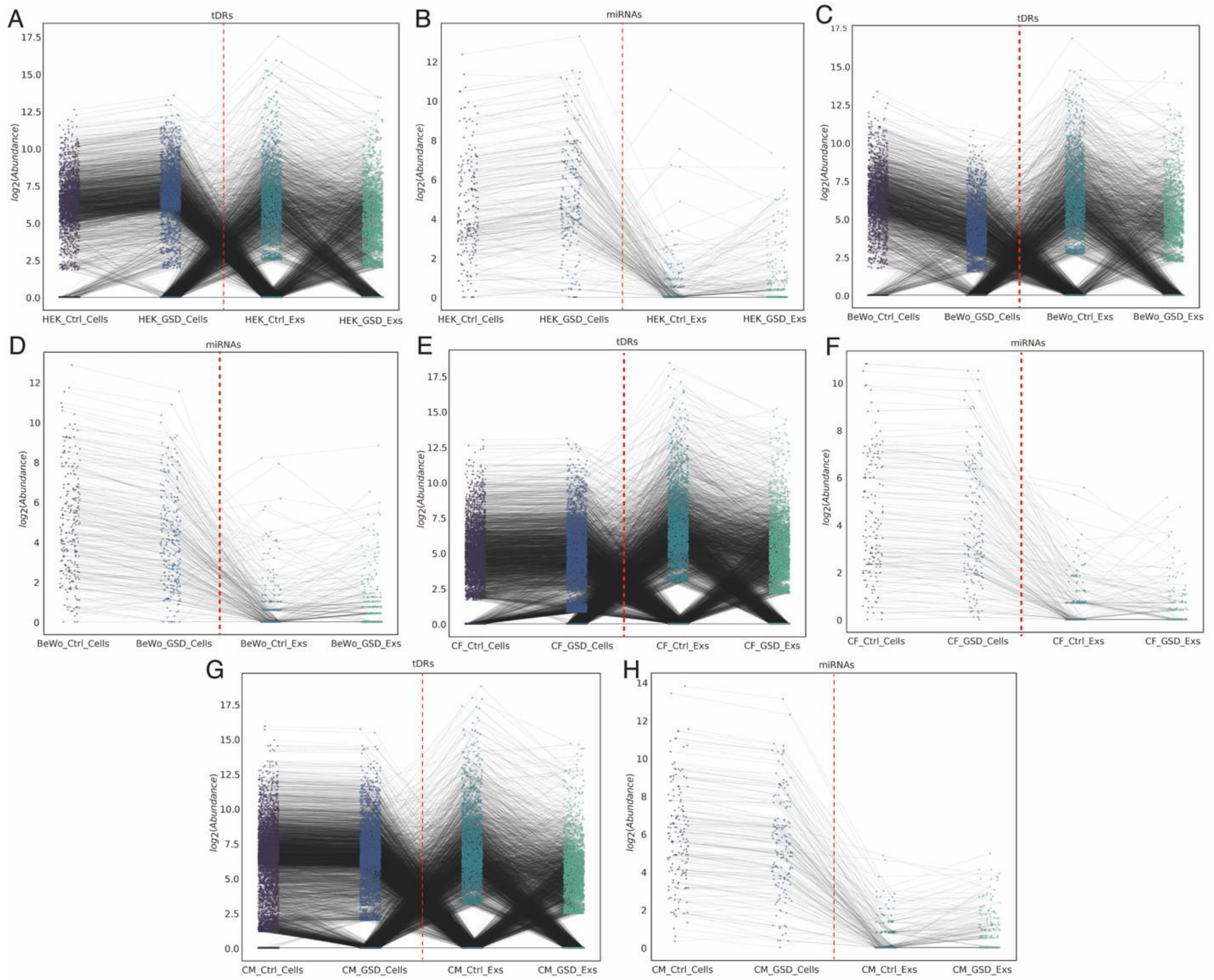

Figure S10. Nutritional deprivation-shaped dynamic expression of cellular and extracellular tDRs and miRNAs

A and **B**. Expression tracing plots reveal more robust changes in tDR expression (A) when compared with miRNA expression (B) in HEK cells during GSD treatment;

C and **D**. Expression tracing plots reveal more robust changes in tDR expression (C) when compared with miRNA expression (D) in BeWo cells during GSD treatment;

E and **F**. Expression tracing plots reveal more dynamic changes in tDR expression (E) when compared with miRNA expression (F) in CF cells during GSD treatment.

G and **H**. Expression tracing plots reveal more dynamic changes in tDR expression (G) when compared with miRNA expression (H) in CM cells during GSD treatment.

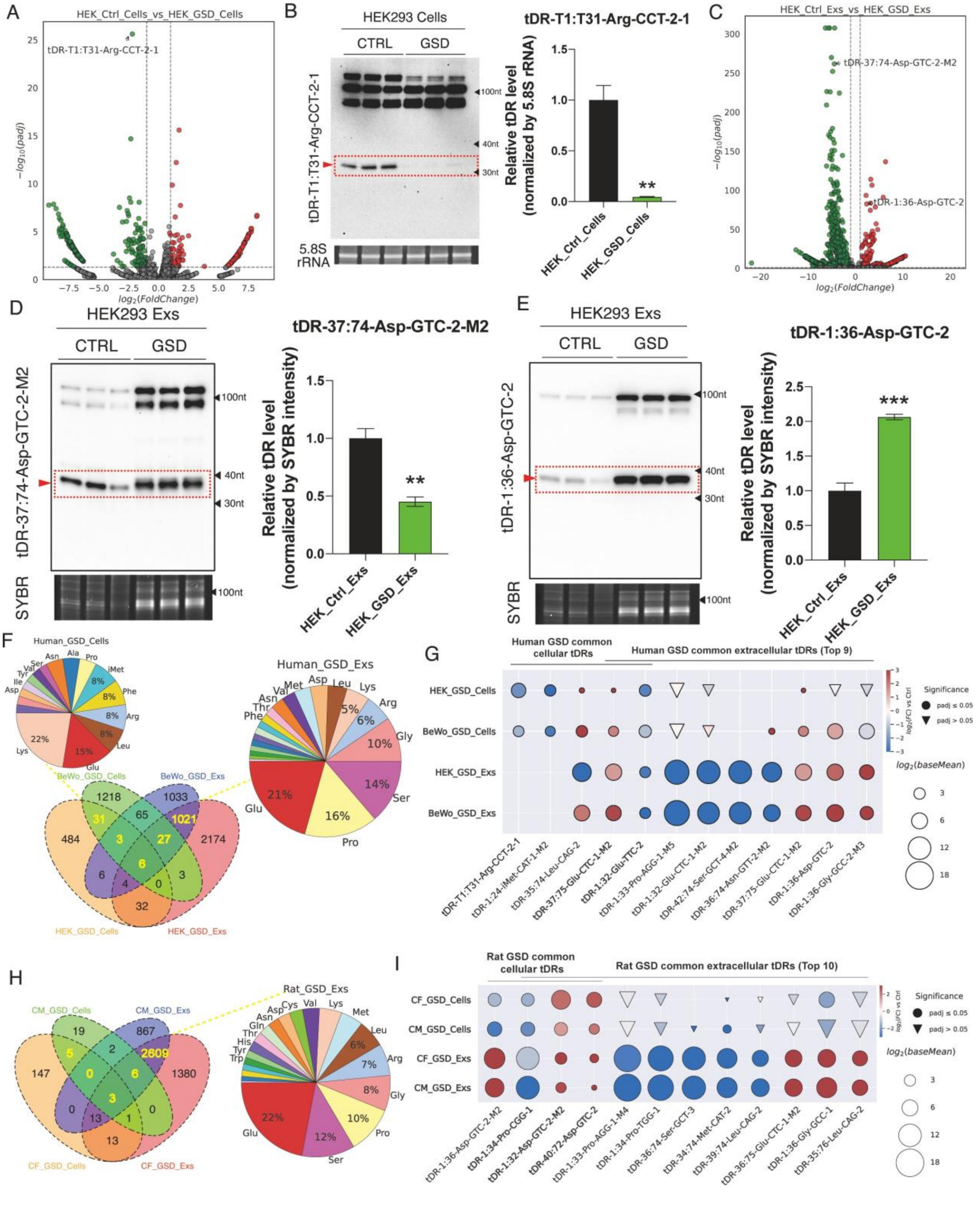

Figure S11. Nutritional deprivation-shaped cellular and extracellular tDR signatures

A. Volcano plot shows the differentially expressed tDRs in HEK cells after GSD treatment.

B. Northern blot validation of the downregulation of tDR-73i1:T19-Arg-CCT-2-1 after GSD treatment in HEK cells.

C. Volcano plot shows the differentially expressed tDRs in the HEK cell-derived Exs after GSD treatment.

D and **E**. Northern blot validation of the downregulation of tDR-37:74-Asp-GTC-2-M2 (D) and the upregulation of tDR-1:36-Asp-GTC-2 in HEK cell-derived Exs after GSD treatment.

F. The number and parent tRNA isotype distribution of the cellular and extracellular tDRs that were regulated by GSD in both HEK and BeWo.

G. The most significant cellular and extracellular tDRs that were regulated by GSD in both HEK and BeWo.

H. The number and parent tRNA isotype distribution of the cellular and extracellular tDRs that were regulated by GSD in both CF and CM.

I. The most significant cellular and extracellular tDRs that were regulated by GSD in both CF and CM.

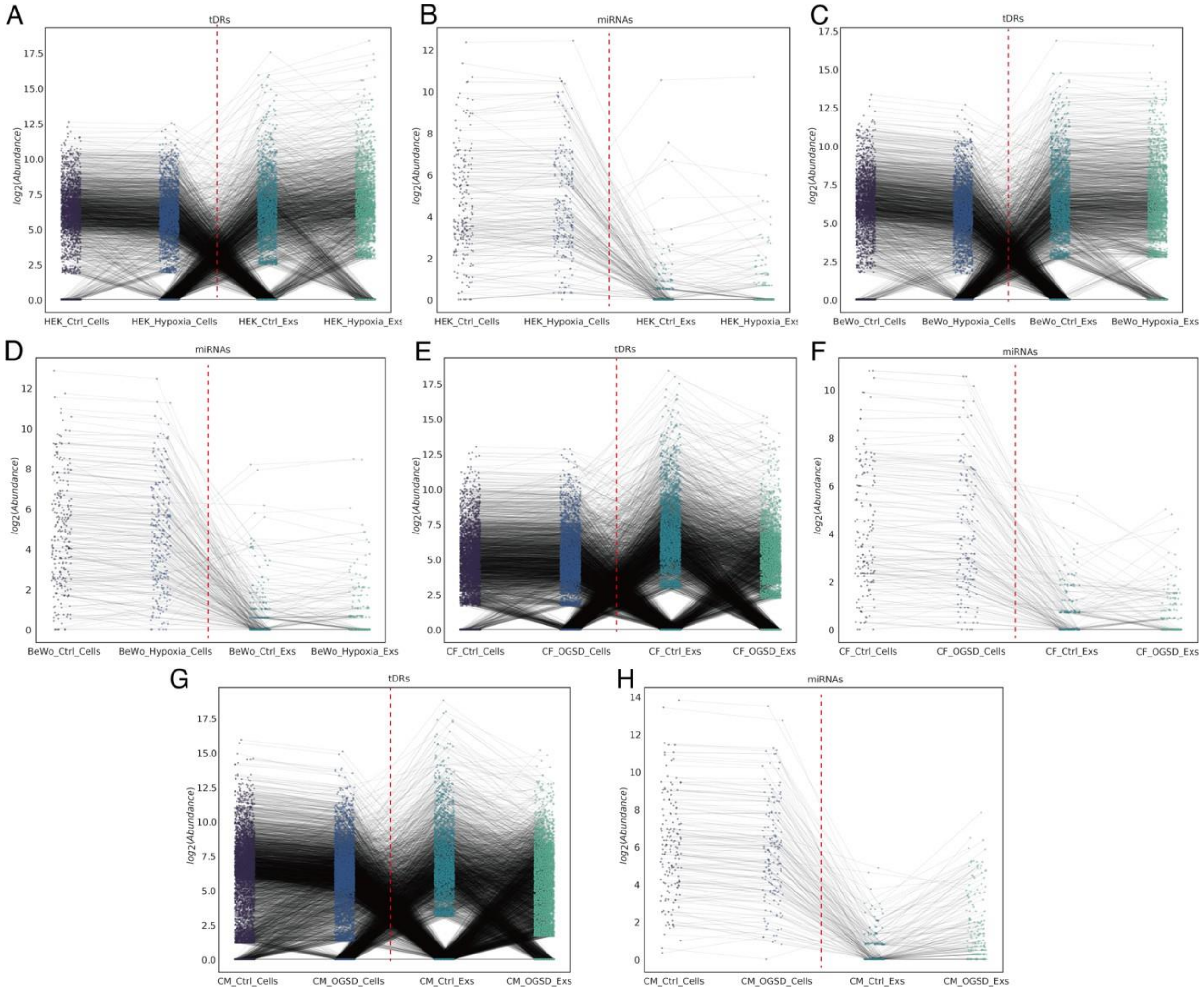

Figure S12. Hypoxia-shaped dynamic expression of cellular and extracellular tDRs and miRNAs

A and **B**. Expression tracking plots reveal more robust changes in tDR expression (A) when compared with miRNA expression (B) in HEK cells during hypoxia treatment;

C and **D**. Expression tracking plots reveal more robust changes in tDR expression (C) when compared with miRNA expression (D) in BeWo cells during hypoxia treatment;

E and **F**. Expression tracking plots reveal more robust changes in tDR expression (E) when compared with miRNA expression (F) in CM cells during hypoxia treatment;

G and **H**. Expression tracking plots reveal more dynamic changes in tDR expression (G) when compared with miRNA expression (H) in CF cells during hypoxia treatment.

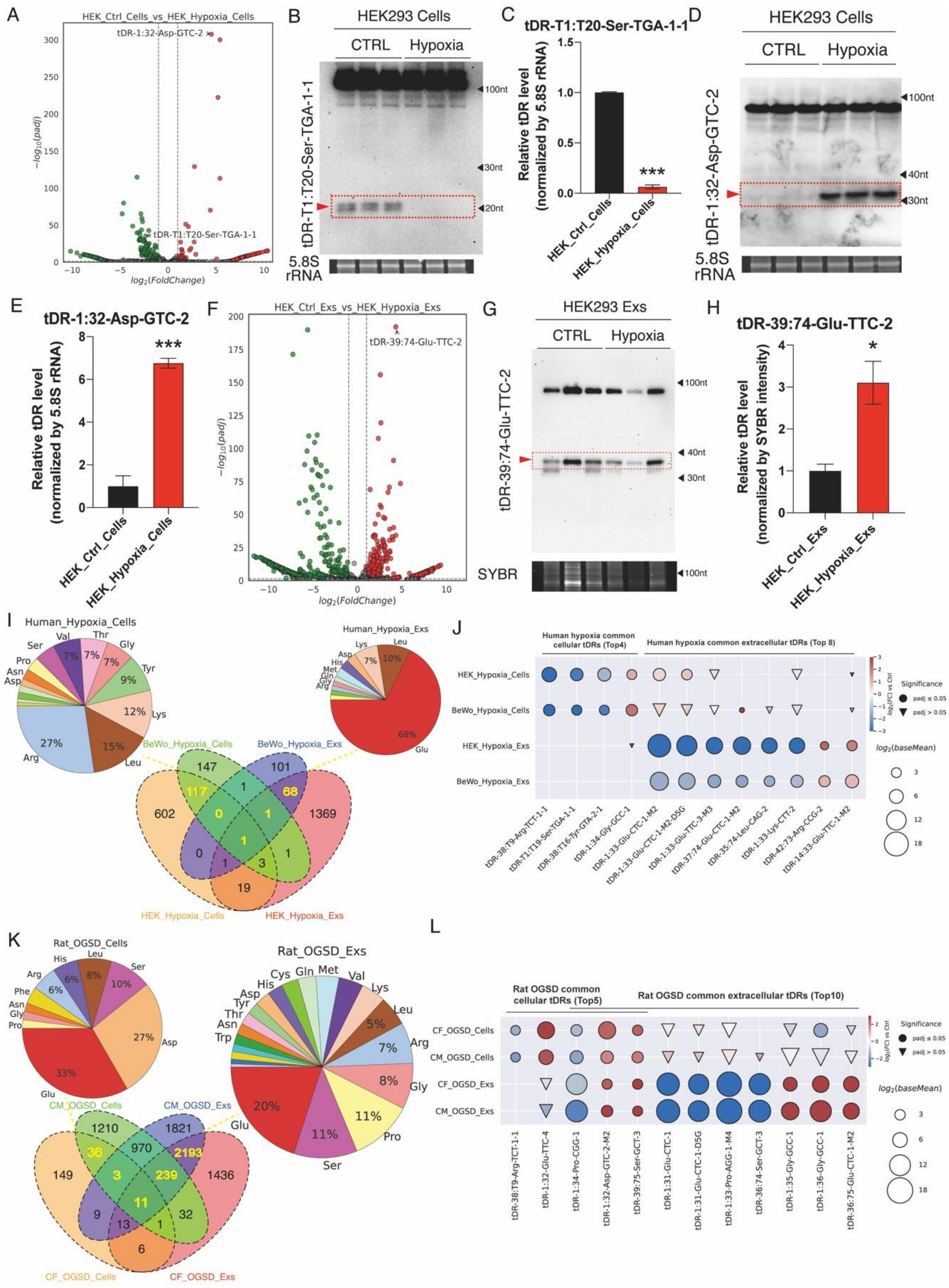

Figure S13. Hypoxia-shaped cellular and extracellular tDR signatures

A. Volcano plot shows the differentially expressed tDRs in HEK cells after hypoxia treatment.

B and **C**. Northern blot validation of the downregulation of tDR-T1:T20-Ser-TGA-1-1 after hypoxia treatment in HEK cells.

D and **E**. Northern blot validation of the upregulation of tDR-1:32-Asp-GTC-2 after hypoxia treatment in HEK cells.

F. Volcano plot shows the differentially expressed tDRs in the HEK cell-derived Exs after hypoxia treatment.

G and **H**. Northern blot validation of the upregulation of tDR-39:74-Glu-TTC-2 in the HEK cell-derived Exs after hypoxia treatment.

I. The number and parent tRNA isotype distribution of the cellular and extracellular tDRs that were regulated by hypoxia in both HEK and BeWo.

J. The most significant cellular and extracellular tDRs that were regulated by hypoxia in both HEK and BeWo.

K. The number and parent tRNA isotype distribution of the cellular and extracellular tDRs that were regulated by OGSD in both CF and CM.

L. The most significant cellular and extracellular tDRs that were regulated by OGSD in both CF and CM.

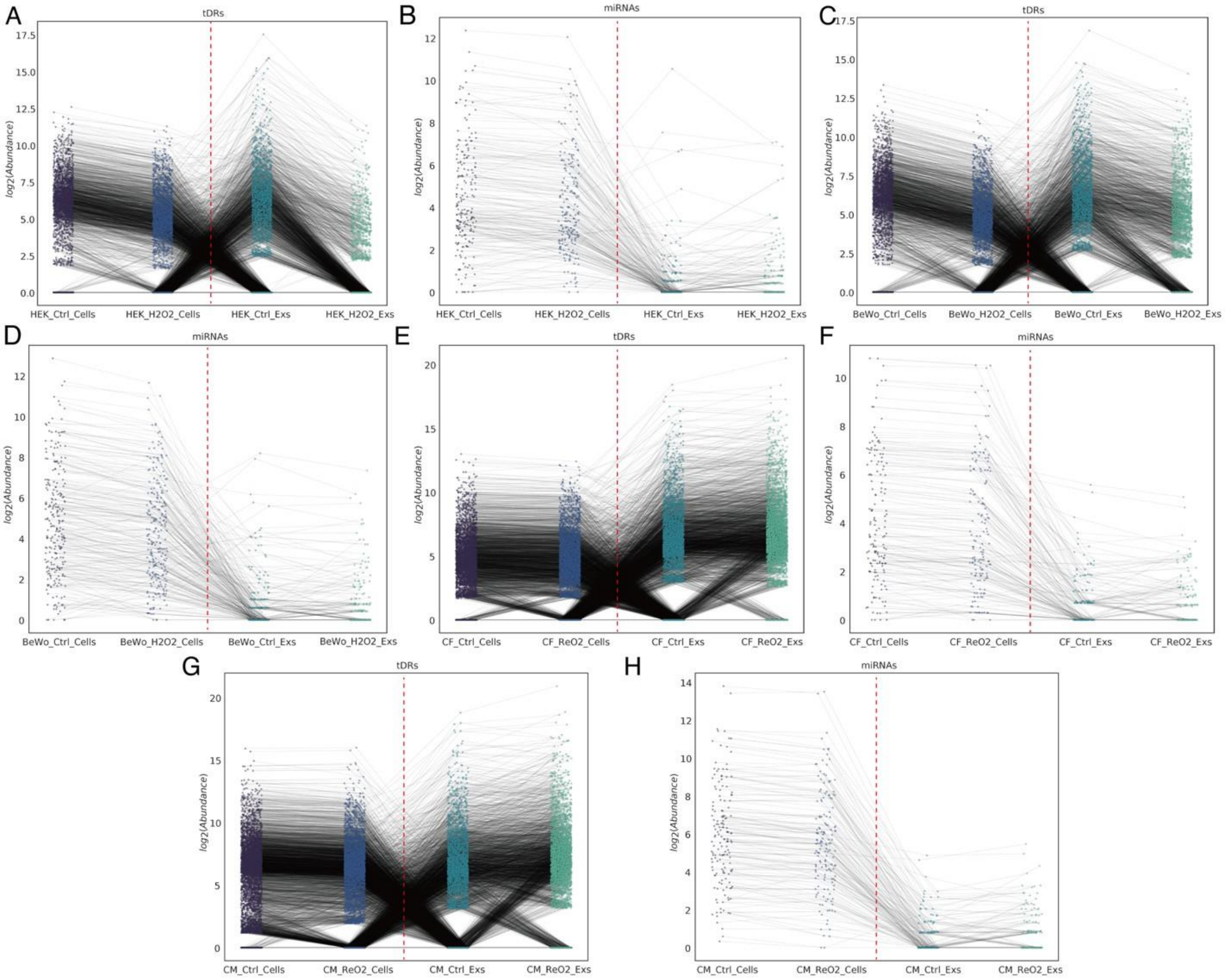

Figure S14. Oxidative stress-shaped dynamic expression of cellular and extracellular tDRs and miRNAs

A and **B**. Expression tracking plots reveal more robust changes in tDR expression (A) when compared with miRNA expression (B) in HEK cells during H_2O_2 treatment;

C and **D**. Expression tracking plots reveal more robust changes in tDR expression (C) when compared with miRNA expression (D) in BeWo cells during H_2O_2 treatment;

E and **F**. Expression tracking plots reveal more robust changes in tDR expression (E) when compared with miRNA expression (F) in CM cells during ReO_2 treatment;

G and **H**. Expression tracking plots reveal more dynamic changes in tDR expression (G) when compared with miRNA expression (H) in CF cells during ReO_2 treatment.

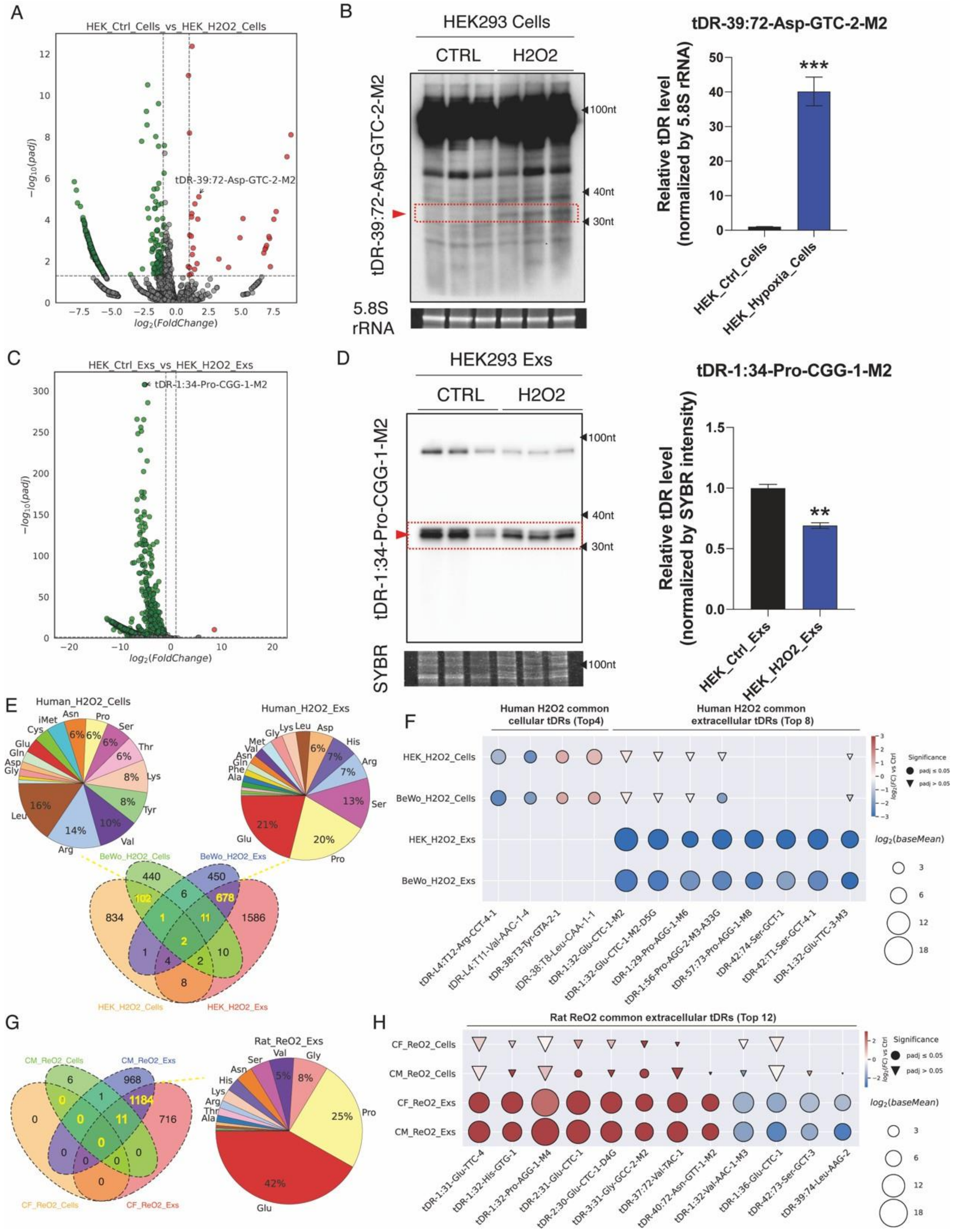

Figure S15. Oxidative stress-shaped cellular and extracellular tDR signatures

A. Volcano plot shows the differentially expressed tDRs in HEK cells after H_2O_2 treatment.

B. Northern blot validation of the upregulation of tDR-39:72-Asp-GTC-2-M2 after H_2O_2 treatment in HEK cells.

C. Volcano plot shows the differentially expressed tDRs in the HEK cell-derived Exs after H_2O_2 treatment.

D. Northern blot validation of the downregulation of tDR-1:34-Pro-CGG-1-M2 in the HEK cell-derived Exs after H_2O_2 treatment.

E. The number and parent tRNA isotype distribution of the cellular and extracellular tDRs that were regulated by H_2O_2 in both HEK and BeWo.

F. The most significant cellular and extracellular tDRs that were regulated by H_2O_2 in both HEK and BeWo cells.

G. The number and parent tRNA isotype distribution of the cellular and extracellular tDRs that were regulated by ReO_2 in both CF and CM.

H. The most significant cellular and extracellular tDRs that were regulated by ReO_2 in both CF and CM.

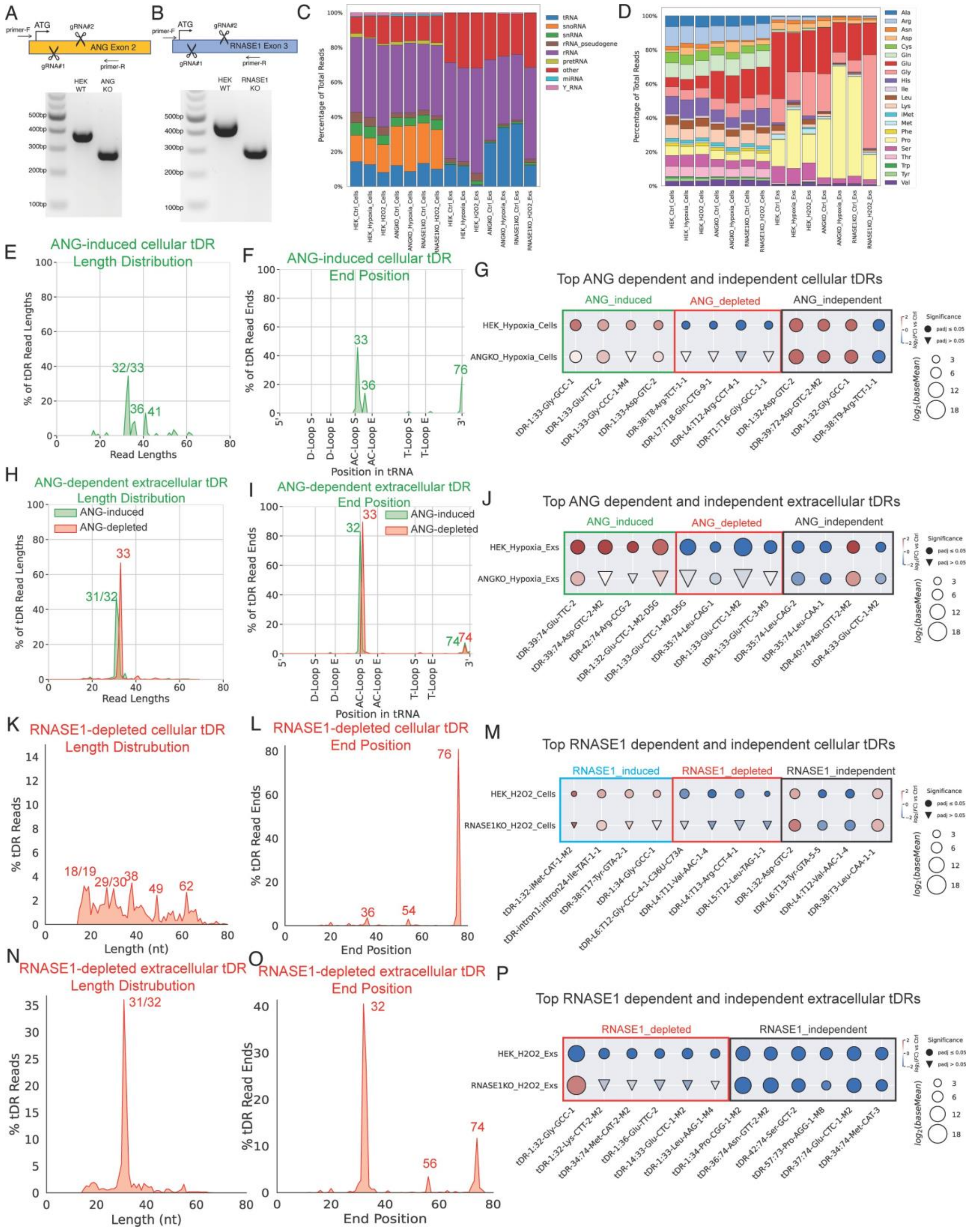

Figure S16. ANG and RNASE1 are critically involved in stress-modulated cellular and extracellular tDR signatures.

A. Genomic DNA PCR validation of the ANG-knockout monoclonal HEK cells.

B. Genomic DNA PCR validation of the RNASE1-knockout monoclonal HEK cells.

C. Both ANG and RNASE1 knockout significantly increased the tRNA/tDR reads in the extracellular samples but not in cellular samples.

D. ANG and RNASE1 knockout dramatically changed the proportion of tRNA-Gly, tRNA-Pro, and tRNA-Ser in the Exs samples.

E. Length distribution of ANG-induced cellular tDRs.

F. End positions of ANG-induced cellular tDRs.

G. The top 4 most significant hypoxia-regulated cellular tDRs that are generated by ANG (green box), are targeted for degradation by ANG (red box), and are independent of ANG (black box).

H. Length distribution of ANG-induced and ANG-depleted extracellular tDRs.

I. End positions of ANG-induced and ANG-depleted extracellular tDRs.

J. The top 4 most significant hypoxia-regulated extracellular tDRs that are generated by ANG (green box), are targeted for degradation by ANG (red box), and are independent of ANG (black box).

K. Length distribution of RNASE1-depleted cellular tDRs.

L. End positions of RNASE1-depleted cellular tDRs.

M. The top 4 most significant H_2O_2-regulated cellular tDRs that are generated by RNASE1 (blue box), are targeted for degradation by RNASE1 (red box), and are independent of RNASE1 (black box).

N. Length distribution of RNASE1-depleted extracellular tDRs.

O. End positions of RNASE1-depleted extracellular tDRs.

P. The top 4 most significant H_2O_2-regulated extracellular tDRs that targeted for degradation by RNASE1 (red box), and are independent of RNASE1 (black box).
